# Transcription Termination and Chimeric RNA Formation Controlled by *Arabidopsis thaliana* FPA

**DOI:** 10.1371/journal.pgen.1003867

**Published:** 2013-10-31

**Authors:** Céline Duc, Alexander Sherstnev, Christian Cole, Geoffrey J. Barton, Gordon G. Simpson

**Affiliations:** 1 College of Life Sciences, University of Dundee, Dundee, Scotland, United Kingdom; 2 James Hutton Institute, Invergowrie, Dundee, Scotland, United Kingdom; University of Cambridge, United Kingdom

## Abstract

Alternative cleavage and polyadenylation influence the coding and regulatory potential of mRNAs and where transcription termination occurs. Although widespread, few regulators of this process are known. The *Arabidopsis thaliana* protein FPA is a rare example of a *trans*-acting regulator of poly(A) site choice. Analysing *fpa* mutants therefore provides an opportunity to reveal generic consequences of disrupting this process. We used direct RNA sequencing to quantify shifts in RNA 3′ formation in *fpa* mutants. Here we show that specific chimeric RNAs formed between the exons of otherwise separate genes are a striking consequence of loss of FPA function. We define intergenic read-through transcripts resulting from defective RNA 3′ end formation in *fpa* mutants and detail cryptic splicing and antisense transcription associated with these read-through RNAs. We identify alternative polyadenylation within introns that is sensitive to FPA and show FPA-dependent shifts in *IBM1* poly(A) site selection that differ from those recently defined in mutants defective in intragenic heterochromatin and DNA methylation. Finally, we show that defective termination at specific loci in *fpa* mutants is shared with *dicer-like 1 (dcl1)* or *dcl4* mutants, leading us to develop alternative explanations for some silencing roles of these proteins. We relate our findings to the impact that altered patterns of 3′ end formation can have on gene and genome organisation.

## Author Summary

The ends of almost all eukaryotic protein-coding genes are defined by a poly(A) signal. When genes are transcribed into mRNA by RNA polymerase II, the poly(A) signal guides cleavage of the precursor mRNA at a particular site; this is accompanied by the addition of a poly(A) tail to the mRNA and termination of transcription. Many genes have more than one poly(A) signal and the regulated choice of which to select can effectively determine what the gene will code for, how the gene can be regulated and where transcription termination occurs. We discovered a rare example of a regulator of poly(A) site choice, called FPA, while studying flower development in the model plant *Arabidopsis thaliana*. Studying FPA therefore provides an opportunity to understand not only its roles in plant biology but also the generic consequences of disrupting alternative polyadenylation. In this study, we use a technique called direct RNA sequencing to quantify genome-wide shifts in poly(A) site selection in plants that lack FPA function. One of our most striking findings is that in the absence of FPA we detect chimeric RNAs formed between two otherwise separate and well-characterised genes.

## Introduction

Eukaryotic mRNA 3′ ends are defined by a protein complex that cleaves pre-mRNA in close association with RNA polymerase II (Pol II) and adds a poly(A) tail to the free 3′ end [1], [2]. This event is closely associated with transcription termination, since cleavage exposes the 5′ end of the nascent RNA to a 5′–3′ exonuclease that degrades the RNA up to the exit channel of Pol II, hence contributing to termination [3]. However, termination is the least understood aspect of the transcription cycle [3] and at a sub-set of mammalian genes, cleavage and polyadenylation occur post-transcriptionally because rapid cleavage of nascent RNA at co-transcriptional cleavage (CoTC) sites downstream of the poly(A) signal promotes termination and the release of pre-mRNA from the chromatin template [4].

The selection of alternative cleavage and polyadenylation sites defines different 3′ ends within pre-mRNAs transcribed from a single gene and can therefore affect function by determining coding potential and the inclusion of regulatory sequence elements. Termination efficiency can also affect transcript levels, possibly because termination facilitates recycling of transcription complexes [5]–[7]. Therefore, the processes of cleavage, polyadenylation and termination are important stages at which gene expression can be regulated. However, the widespread nature of this control has only become apparent relatively recently [1], [2].

A less well-studied phenomenon, which suggests an additional role for regulated 3′ end formation and transcription termination, is the existence of chimeric transcripts formed between exons of neighbouring genes encoded on the same chromosomal strand [8]–[10]. Specific chimeric RNAs are conserved in vertebrates [10], regulated under certain conditions [11] and occur recurrently in cancerous tissues [12], [13]. However, regulatory processes controlling chimeric RNA formation are poorly understood.

The spen family protein FPA is a *trans*-acting regulator of RNA 3′ end formation, but is not a conserved component of either the splicing or the cleavage and polyadenylation apparatus [14]. First identified as a regulator of flower development, FPA enables flowering by ultimately limiting the expression of the floral repressor FLC [15]. Intriguingly, several viable *A. thaliana* mutants disrupted in factors that mediate RNA 3′ end formation are late flowering as a result of the specific misregulation of *FLC* [16]–[18]. The mechanism by which FPA controls *FLC* is unknown, but increased *FLC* transcription in *fpa* mutants is accompanied by increased levels of alternatively processed non-coding antisense RNAs (asRNAs) at the *FLC* locus [14]. The biological role of FPA is not restricted to flowering, since FPA also influences other aspects of development [19], [20]. In addition, *fpa* mutants were isolated in a screen designed to identify factors required for RNA silencing [21]. However, the involvement of FPA in endogenous RNA silencing pathways is currently unclear because the apparent misregulation of an endogenous RNA silencing target (the SINE retroelement *AtSN1*) in *fpa* mutants can be explained by read-through resulting from defective 3′ end formation at an upstream gene [14].

There is intense interest in determining how the widespread alternative patterns of RNA 3′ end formation can be regulated and what the consequences of disrupting specific regulators may be. We recently defined genome-wide patterns of cleavage and polyadenylation in *A. thaliana* using direct RNA sequencing (DRS), thereby refining our understanding of 3′ end formation in this model organism [22]. DRS can define the site of RNA cleavage and polyadenylation with an accuracy of ±2 nt in the absence of errors induced by reverse transcriptase, internal priming, ligation or amplification [22], [23]. In this study, we set out to answer two questions by quantifying shifts in RNA 3′ end formation between wild-type (WT) and *fpa* mutants with DRS: (1) could we clarify the roles of FPA in plant biology (particularly in relation to flowering and RNA silencing)?; and (2) could we define generic consequences of defective RNA 3′ end regulation that would be of broad relevance? Here we identify the abundance and sites of 3′ end formation of RNAs transcribed antisense to the floral regulator *FLC*, but do not detect evidence of a widespread role for FPA in RNA-mediated chromatin silencing. We identify the generic consequences of disrupting regulated RNA 3′ end formation, prominent among which is the formation of specific chimeric RNAs between exons of otherwise separate and well-characterised genes. In addition, we make the unexpected but related discovery that transcription termination defects in *fpa* are shared at some of the same loci in both *dcl1* and *dcl4* mutants. Consequently, we suggest an alternative explanation involving defective upstream termination for the previously reported DCL1-mediated silencing of overlapping gene pairs [24].

## Results

### Loss of FPA function alters patterns of gene expression

**Figure 1:**
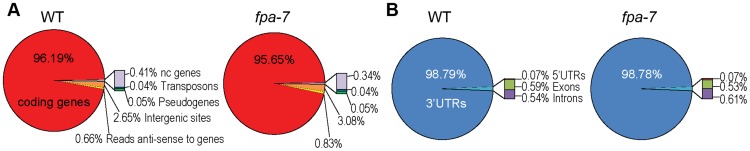
Distribution of DRS reads. (*A*) Genome-wide distribution after re-annotation in wild-type (WT) and *fpa-7*. (*B*) Distribution of DRS reads mapping to protein-coding genes after re-annotation.

We subjected total RNA purified from three biological replicates of WT *A. thaliana* [Columbia-0 (Col-0) accession] and *fpa-7* loss-of-function mutants to DRS. RNA was prepared from 14-day-old whole seedlings. A total of 22,560,508 WT and 24,383,585 *fpa-7* reads that map polyadenylated RNA 3′ ends were aligned uniquely to the most recent *A. thaliana* genome release (currently TAIR10). A summary of the read statistics is given in Table S1. In each genotype, the vast majority of reads mapped to 3′ untranslated regions (UTRs) of protein-coding genes (Figure 1A–B). The DRS data can be visualised using aligned reads available at www.compbio.dundee.ac.uk/polyADB/.

**Figure 2:**
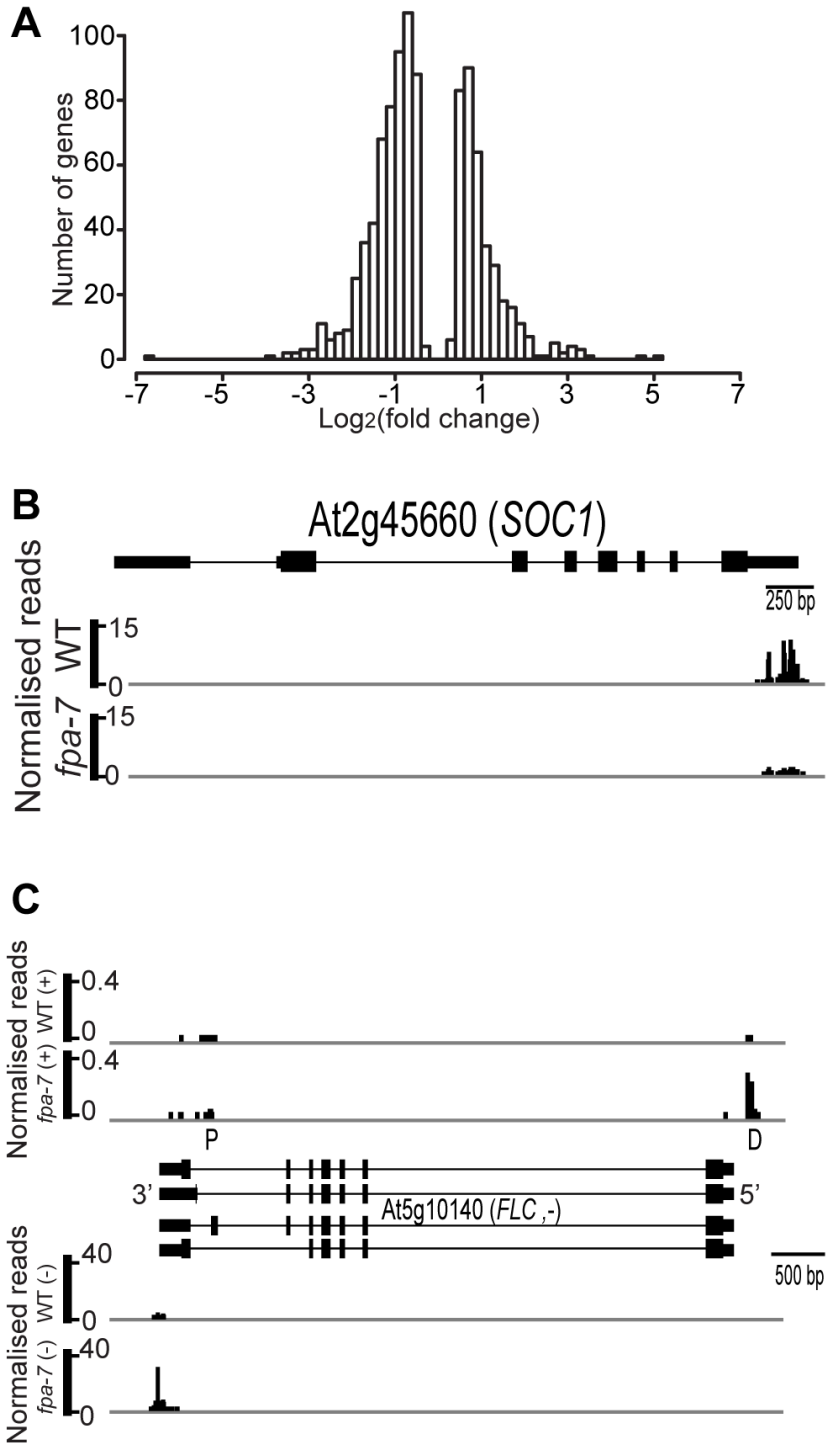
Differentially expressed genes between wild-type and *fpa-7*. (*A*) Histogram of log_2_ fold change profiles for protein-coding genes differentially expressed (DE) between wild-type (WT) and *fpa-7*. (*B*) Reads mapping to the locus encoding SO*C1*. The *SOC1* gene is orientated 5′–3′. (*C*) Reads mapping to the locus encoding *FLC*. Normalised reads are presented for WT and *fpa*. The top panel displays the reads corresponding to the *FLC* asRNAs and corresponds to the (+) strand while the bottom panel displays the reads corresponding to *FLC* mRNA and corresponds to the (−) strand. The *FLC* gene is orientated 3′–5′, while the reads corresponding to *FLC* asRNAs are orientated 5′–3′. Proximally polyadenylated ‘P’ *FLC* asRNAs were detected in *fpa-7* and WT by the low number of reads at surrounding sites, while the distal ‘D’ cleavage sites were clearly defined. Exons are denoted by rectangles, UTRs by adjoining narrower rectangles and introns by lines. Images of normalised read alignments were made using the Integrated Genome Browser [55] and correspond to combined reads from the three sequenced biological replicates for each genotype.

We first asked whether DRS could reveal changed patterns of gene expression between genotypes by measuring the difference in read counts mapped to annotated protein-coding genes. We used DESeq [25] to detect differential gene expression between the WT and *fpa* mutant DRS datasets. The expression of 18,406 protein-coding genes was detected in WT *A. thaliana*; DESeq analysis suggested that 1,114 genes were differentially expressed in the *fpa-7* mutant (Table S2), with the vast majority being up- or down-regulated by less than two-fold (Figure 2A). Since *fpa* mutants flower late, a number of gene expression changes in *fpa* are either predictable or already established. For example (and consistent with our expectations), down-regulation of the floral pathway integrator *SUPPRESSOR OF OVEREXPRESSION OF CONSTANS* 1 (*SOC1*; fold change = 0.13, *P* = 10e^−96^) and up-regulation of the floral repressor *FLC* (fold change = 27, *P* = 3.10e^−139^) were readily detected (Figures 2B–C and S1A).

### *fpa* mutants contain increased numbers of asRNA transcripts cleaved antisense to the *FLC* promoter

We previously reported that increased read-through of asRNA transcripts through the *FLC* locus in *fpa* mutants correlates with increased sense strand transcription [14]; this seemingly counterintuitive finding was confirmed here by DRS (Figures 2C and S1A). DRS identified the preferred sites of asRNA cleavage and polyadenylation (Figure 2C), indicated that asRNA expression is approximately 100-fold lower than *FLC* sense strand expression (Figure 2C) and showed that, in *fpa* mutants, increased levels of sense strand *FLC* expression are associated with increased levels of asRNAs cleaved and polyadenylated antisense to the *FLC* promoter (Figures 2C and S1A). In contrast to another report [18], DRS data did not indicate reduced levels of cleavage and polyadenylation at proximal sites in the asRNAs in *fpa* mutants, although this interpretation would benefit from a greater sequencing depth (Figure 2C). A single nucleotide polymorphism (SNP) associated with variation in flowering time and asRNA expression level [26] maps to the distal poly(A) signal of preferred cleavage sites antisense to the *FLC* promoter (Figure S1B). These DRS data clarify multiple 3′ end processing events at the *FLC* locus for the first time and are therefore valuable for understanding how the recurrent identification of 3′ end processing factors might affect *FLC* expression [14], [16]–[18].

### FPA does not play a widespread role in RNA-mediated chromatin silencing

**Figure 3:**
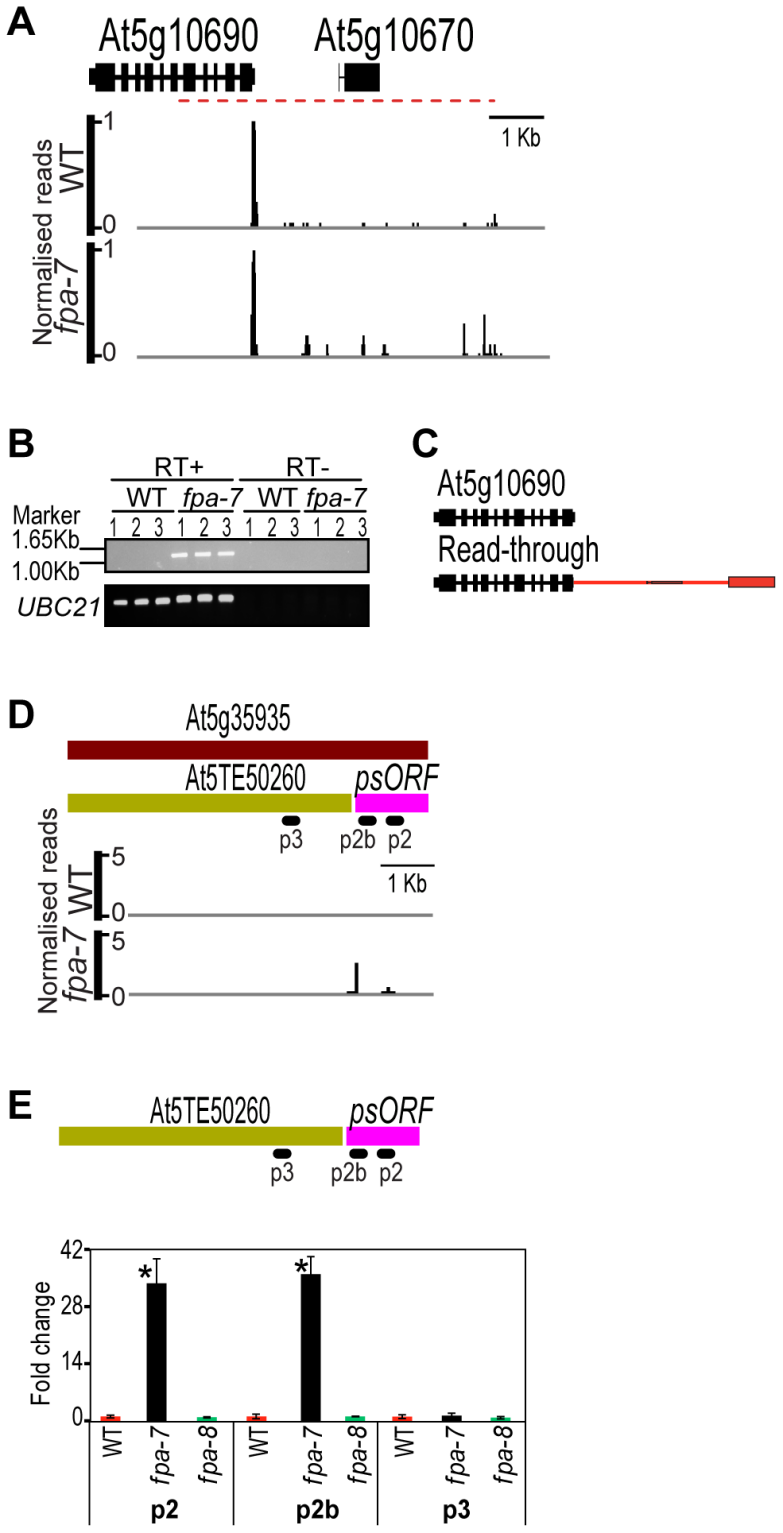
Differentially expressed transposons between wild-type and *fpa-7*. (*A*) Differential expression of the transposable element gene (At5g10670) in *fpa-7*. (*B*) Read-through contiguous RNAs were validated by RT-PCR (red dashed line). Three biological replicates (1, 2 and 3) were used for each genotype: wild-type (WT) and *fpa*-7. UBIQUITIN *LIGASE 21* (*UBC21*) was used as a control. RT-PCR products were separated on agarose gels and stained with ethidium bromide. (*C*) Transcripts are either cleaved and polyadenylated in the annotated 3′UTR or at the intergenic sites, as determined by sequencing the cloned RT-PCR products. Red rectangles represent the 3′UTR specific to the read-through transcript and red lines represent 3′UTR introns. (*D*) Differential expression of the transposable element gene (At5g35935) in *fpa-7*. Recent re-annotation of At5g35935 [12], [13], [28], [29] defines two transcription units within it: the recently arisen pseudogene *psORF* and the transposon At5TE50260. DRS data reveal that silencing of *psORF* is lost in *fpa-7*. (*E*) RT-qPCR analysis of *psORF* in *fpa-7* and *fpa-8* mutant alleles. Silencing of *psORF* (p2 and p2b) is lost in *fpa-7* but not in *fpa-8*. Data are the means ± SEM obtained for three independent PCR amplifications of three biological replicates. The y-axis shows the fold change relative to WT (WT set to 1) after normalisation to *UBC21* gene expression. Location of the RT-qPCR amplicon is displayed on the left panel. *, *P*<0.05; Student's t-test. Normalised reads mapping to the different loci are presented for WT and *fpa*. Genes are orientated 5′–3′; exons are denoted by rectangles, UTRs by adjoining narrower rectangles and introns by lines. Images of normalised read alignments were made using the Integrated Genome Browser [55] and correspond to combined reads from the three sequenced biological replicates for each genotype.

Although FPA has been reported to play a widespread role in RNA-mediated chromatin silencing [21], we found statistically significant increases in *fpa-7* DRS counts at only 28 of the 31,189 transposons and 3,903 transposable element genes annotated in TAIR10 (Table S3). Since we had previously found that the apparent involvement of FPA in silencing the SINE retroelement *AtSN1* could be explained by read-through resulting from defective termination at an upstream Pol II gene [14], [21], we asked whether reads mapping to annotated transposons reflect a genuine loss of silencing or whether they could also be explained by read-through. We found, for example, that DRS reads mapping to an apparently up-regulated transposable element gene (At5g10670) did indeed result from read-through from the upstream protein-coding gene At5g10690 (Figure 3A–C). Therefore, at least some of the relatively small number of reads mapping to transposons in this study may also be explained by read-through events. Clearly, not all misregulated transposons will be polyadenylated and so they will not be detected here, but the results of this genome-wide analysis are inconsistent with the suggestion that FPA plays a widespread role in RNA-mediated chromatin silencing. This conclusion is supported by a recent DNA methylation analysis of *A. thaliana* silencing mutants that included *fpa-7* and found no evidence of FPA affecting RNA-dependent DNA methylation (RdDM) target sites [27].

The number of DRS reads mapping to the transposable element At5g35935 was significantly different between WT and *fpa-7* (Figure 3D; fold change = 33, *P* = 2.63e^−32^). Recent re-annotation of At5g35935 defined a newly arisen pseudogene *psORF* (pseudogene small open reading frame) within this sequence [28], [29], and it is the increased expression in *fpa-7* of *psORF*, rather than the transposon, that is detected by DRS (Figure 3D–E). According to recently published DNA methylation data [27], *psORF* is demethylated in *fpa-7* mutants (Figure S2A), and we found no evidence that read-through from an upstream gene could account for the increased number of DRS reads detected here in *fpa-7*. These findings therefore raised the possibility that FPA functions to silence this newly arisen pseudogene. However, we did not detect misregulation of *psORF* in a second allele, *fpa-8* (Figure 3E). It has recently been shown that *de novo* originated *A. thaliana* genes might be prone to epigenetic variation in the early stages of their formation [30]. Accordingly, apparent changes in the DNA methylation or expression of such sequences may be a coincidence of the different genetic backgrounds analysed [28], [29]. Such epigenetic variation might also explain why misregulation of antisense RNAs at the newly acquired helitron transposable element At1TE93275 [28], previously reported in *fpa* mutants [31], was not detected in this *fpa-7* dataset (Figure S2B). Consequently, particularly careful analysis is required for the identification of authentic factors mediating the silencing of such newly originated sequences.

### FPA affects intronic cleavage site selection and intergenic read-through

**Figure 4:**
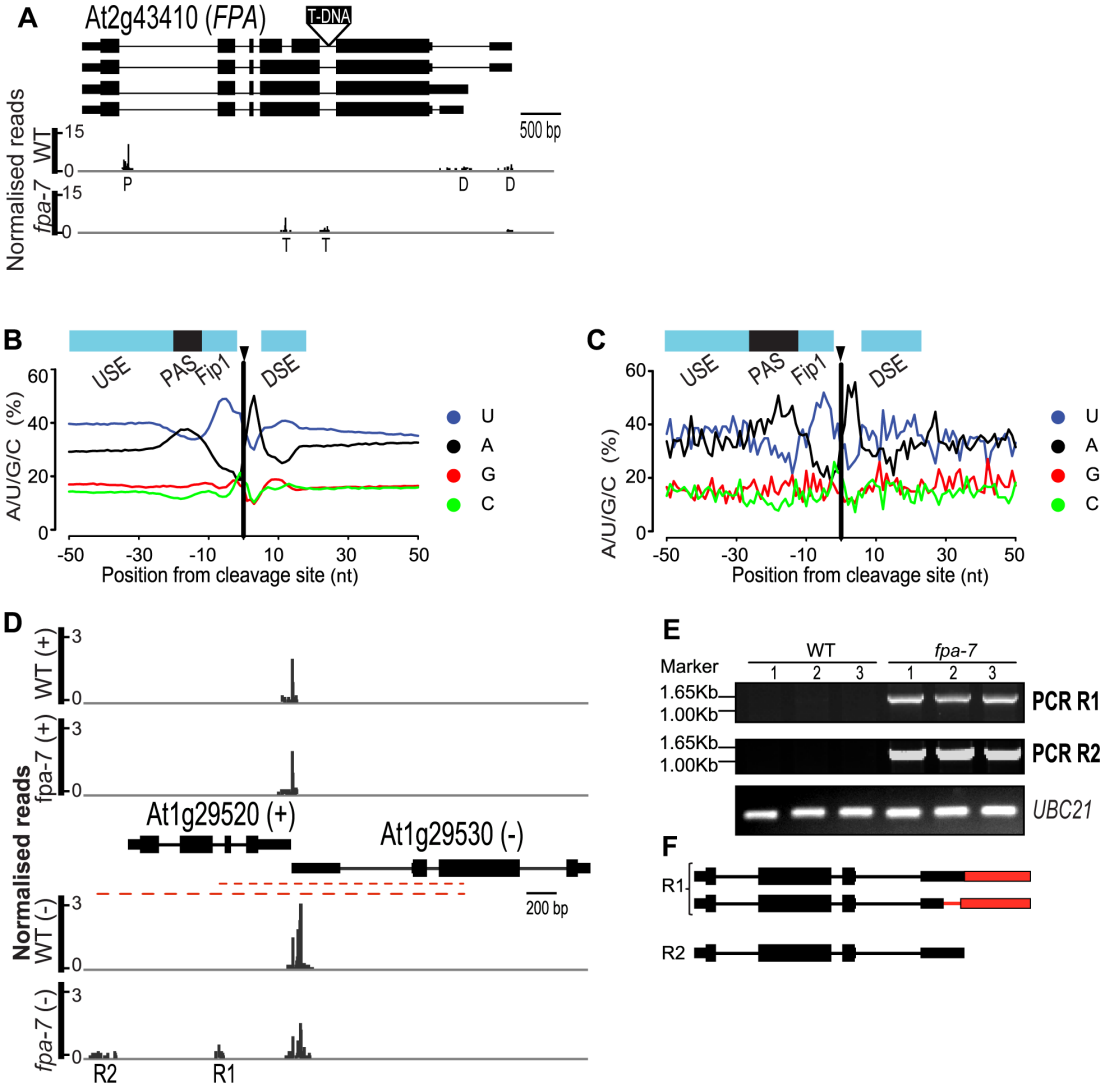
FPA affects intronic cleavage site selection and intergenic read-through. (*A*) Reads mapping to the locus encoding *FPA*. Promoter proximal ‘P’ and distal ‘D’ alternative poly(A) sites are indicated, as are the poly(A) sites ‘T’ resulting from the T-DNA insertion in *fpa-7*. (*B, C*) Nucleotide composition profiles around cleavage sites within annotated genes (*B*) and at intergenic sites (*C*) display alternating A- and U-rich sequences. USE, upstream sequence element; PAS, poly(A) signal; Fip1, the U-rich sequence upstream of the cleavage site is the proposed binding site of FIP1 [58]; DSE, downstream sequence element. (*D–F*) Example of intergenic DRS reads mapping antisense to a coding gene. Normalised reads mapping to the At1g29520–At1g29530 loci are displayed in (*D*). The upper panel shows reads mapping to the (+) strand 3′ end of At1g29520, while the lower panel shows reads mapping to the (−) strand 3′ end of At1g29530. (*E*) R1 and R2 contiguous RNAs were validated by RT-PCR (red dashed lines) with poly(A)+ RNA. RT-PCR products were separated on agarose gels and stained with ethidium bromide. Three biological replicates (1, 2 and 3) were used for each genotype: wild-type (WT) and *fpa*-7. (*F*) Transcripts are either cleaved and polyadenylated in the annotated 3′UTR or at the intergenic sites, as determined by sequencing the cloned RT-PCR products. Red narrower rectangles represent regions specific to the read-through transcript and red lines the 3′UTR introns. Images of normalised read alignments were made using the Integrated Genome Browser [55] and correspond to combined reads from the three sequenced biological replicates for each genotype.

Besides refining our understanding of previously proposed roles for FPA in flowering and RNA silencing, we sought to identify the generic consequences of disrupting a regulator of RNA 3′ end formation. For example, one might predict that 3′ end formation within intronic sites and conventional 3′UTRs would be altered in *fpa* mutants. Cleavage and polyadenylation within intronic sequences outside the 3′UTR can have profound consequences on gene function by truncating mRNA coding potential. FPA effects autoregulation in this way by mediating selection of the promoter proximal intronic cleavage site within its own pre-mRNA [14]. We therefore asked whether FPA controls alternative polyadenylation at other intronic sites. We applied our data-smoothing and peak-finding algorithms to define cleavage sites [22] and estimated differential usage of these sites using DESeq. The validity of this approach was supported by the finding that previously identified intronic alternative polyadenylation events within *FPA* (Figure 4A; *P* = 7.10e^−11^), but not *FCA* were dependent on FPA function [14], [22]. Unexpectedly, additional intronic cleavage sites were also detected in *FPA* RNA itself, but only in the transfer (T)-DNA-induced *fpa-7* allele (Figure 4A) and not in ethyl methanesulfonate (EMS)-induced alleles such as *fpa-8* [21], suggesting that T-DNA insertions can trigger cryptic cleavage and polyadenylation (Figure S3A–F). These allele-dependent distinctions in patterns of *FPA* polyadenylation were indicated by previous RNA gel blot hybridisations [14]. Reduced selection of intronic cleavage sites in 13 genes and increased selection of intronic cleavage sites in another 25 genes were found in *fpa-7* (Table S4), indicating that FPA ultimately promotes cleavage at some sites, but represses it at others.

In order to validate this data analysis, we investigated some of the deduced intronic alternative polyadenylation changes in more detail (Figure S4). For example, we detected shifts in alternative polyadenylation at *IBM1* (*Increase in BONSAI Methylation 1*), a gene encoding a histone demethylase specific for H3K9 dimethylation and monomethylation [32]. Expression of the active enzyme was recently shown to be controlled by alternative polyadenylation, which in turn is dependent on DNA methylation at this locus (Figure S4A) [33] since 3′ end formation occurs exclusively at proximal poly(A) sites in mutants that disrupt CG and CHG DNA methylation [33]. In contrast, our DRS data suggest that cleavage occurs almost exclusively at the *IBM1* distal site in *fpa* mutants (Figure S4B; *P* = 0.03); this was confirmed by RNA gel blot hybridisation (Figure S4C). FPA and DNA methylation mediated by METHYLTRANSFERASE 1 (MET1) and the plant-specific chromomethylase CMT3 therefore appear to have opposing effects on poly(A) site choice in *IBM1* pre-mRNA transcribed through intragenic heterochromatin.

We previously showed that FPA affects 3′ end formation not only within introns but also at conventional 3′UTRs, thus causing intergenic read-through of Pol II transcripts [14]. This discovery was recently extended by a tiling array analysis of *fpa fca* double mutants [31]. Consistent with these previous studies, our DRS analysis mapped reads to intergenic sequences (defined here as regions between protein-coding genes) in *fpa* mutants. DESeq analysis identified 61 up-regulated intergenic regions downstream of down-regulated genes and 109 up-regulated intergenic regions downstream of genes with unchanged expression in *fpa-7* (Table S5). The amount of polyadenylated read-through RNAs relative to upstream annotated genes varied (Figure S5A). We validated potential intergenic read-through events in detail (Figures S5B–Q and S6) and identified three different types of events: first, extended 3′UTRs (Figure S5F–N); second, cryptic splicing event(s) generating altered 3′UTR sequences (Figure S6); and third, read-through accompanied by cryptic splicing that alter the protein-coding sequence of the upstream gene, revealing that intergenic read-through is not necessarily benign (Figure S5O–Q).

DRS extends previous studies by identifying the cleavage and polyadenylation sites of read-through RNAs. We analysed the sequence features of intergenic cleavage sites and, although the relatively small size of the *fpa* read-through dataset makes the nucleotide profile surrounding intergenic cleavage sites appear somewhat noisy, an alternating pattern of A- and U-rich regions flanking the cleavage site and a prominent A-rich region around −20 was detected (Figure 4B–C). We previously showed that the distinguishing feature of preferred and non-preferred cleavage sites within the same 3′UTR is the relative prominence of the A-rich peak located approximately 20 nt upstream of the cleavage site [22] (Figure 4B). Because the corresponding A-rich peak is prominent here, we suggest that 3′ end formation of read-through RNAs in *fpa* mutants takes place at strong poly(A) signals located within intergenic regions.

We identified 14 read-through RNAs in *fpa* mutants that result in transcription of novel RNAs antisense to expressed protein-coding genes (Table S6), but in no case was this associated with down-regulation of sense strand mRNA expression. For example, although we could validate read-through from At1g29530 that generated new antisense RNA against the sense strand-encoded At1g29520, there was no change in the sense strand expression of At1g29520 (Figure 4D–F). Since increased expression of RNA cleaved and polyadenylated antisense to the *FLC* promoter correlates with increased sense strand *FLC* mRNA expression, we next asked whether other genes differentially expressed in *fpa-7* are associated with increased detectable cleavage and polyadenylation of RNA antisense to their promoters. This analysis (Table S7) identified four cases (including *FLC*) in which sense strand gene expression was increased and 13 cases in which it was decreased when increased DRS reads mapping antisense to promoters in *fpa-7* were detected. Overall, we conclude that novel asRNAs generated in *fpa* mutants are not necessarily associated with gene silencing. However, we also identify loci where detailed experimental analysis is required to uncover potentially distinct regulatory consequences of novel asRNAs.

### FPA affects chimeric RNA formation

**Figure 5:**
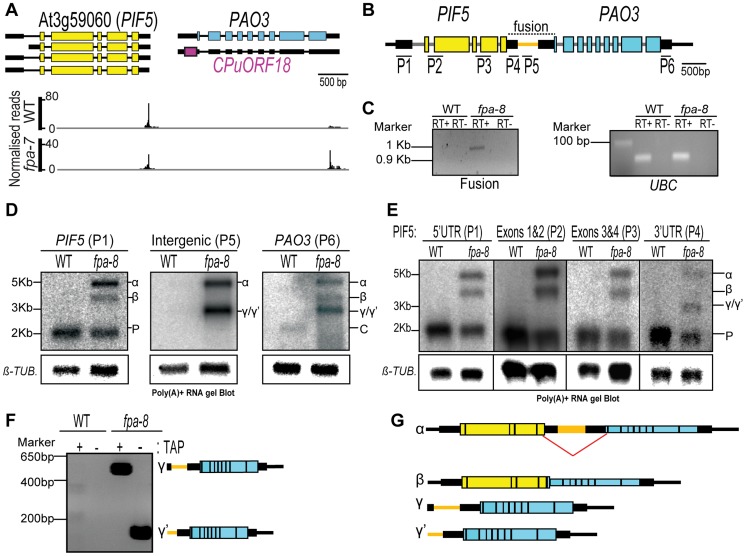
*PIF5–PA03*, an example of chimeric RNA formation controlled by FPA. (*A*) Normalised reads mapping to the locus encoding *PIF5–PA03*. Exons are denoted by coloured rectangles, UTRs by adjoining narrower rectangles and introns by lines. The image of normalised read alignments was made using the Integrated Genome Browser [55] and corresponds to combined reads from the three sequenced biological replicates for each genotype. (*B*) Location of RNA gel blot probes are indicated by numbers (P1–P6) and the tested fusion region by a dotted line. (*C*) RT-PCR analysis of a contiguous RNA between *PIF5* and *PA03* detected in *fpa-8*. *UBIQUITIN LIGASE 21* (*UBC*) was used as a control. (*D,E*) RNA gel blot analysis of *PIF5–PA03* chimeric RNAs in wild-type (WT) and *fpa-8*. P, *PIF5*; C, *PA03* transcripts. *β-TUBULIN* (*β-TUB.*) was used as an internal control. Probes used are shown in (*B*). (*F*) 5′RACE analysis of the γ and γ′ RNAs with or without tobacco acid pyrophosphatase (TAP) treatment. PCR products were separated on an agarose gel and stained with ethidium bromide. (*G*) Schematic representation of the different RNAs expressed at the *PIF5* locus in *fpa* mutants. The splicing event occurring in the β chimeric RNA is shown by a red line.

**Figure 6:**
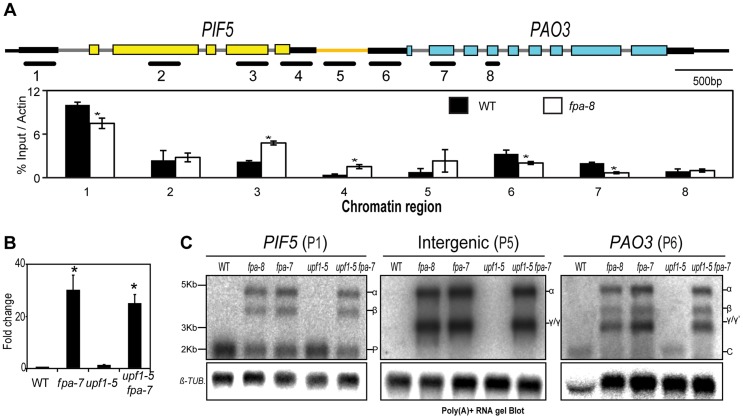
*PIF5–PA03*, an example of chimeric RNA formation controlled by FPA. (*A*) H3K4me3 Chromatin ImmunoPrecipitation (ChIP) analysis of genomic regions at the *PIF5* locus. γ RNAs result from a shift in the transcription start site. Black lines depict the chromatin regions analysed by qPCR. Histograms show means ± SEM obtained for enrichment calculated by percentage input normalised against actin for three PCR amplifications. *, *P*<0.05; Student's t-test. (*B*) RT-qPCR analysis of *PIF5–PA03* chimeric RNAs in *fpa* and *upf1* mutants. Data are the means ± SEM obtained for three independent PCR amplifications of three biological replicates. The y-axis shows the fold change relative to wild-type (WT; (WT set to 1) after normalisation to *UBC21* gene expression. Location of the RT-qPCR amplicon is displayed in Figure S7 *D*. *, *P*<0.05; Student's t-test. (*C*) RNA gel blot analysis of *PIF5–PA03* chimeric RNAs in *fpa* and *upf1* mutants.

Among those RNAs affected by FPA, we identified cases in which tandem genes showed reciprocal changes in read abundance. For example, in the *fpa-7* mutant we found a reduction in the number of reads aligning to the 3′ end of At3g59060 (*PHYTOCHROME INTERACTING FACTOR 5, PIF5*) compared to WT, but the number of reads mapping to the 3′ end of the downstream gene At3g59050 (*POLYAMINE OXIDASE 3, PA03*) was increased (Figure 5A). We asked whether these read differences could be explained by defective 3′ end formation at *PIF5* in *fpa* mutants, leading to chimeric RNAs being cleaved and polyadenylated within the 3′UTR of *PA03*. Consistent with this idea, RT-PCR analysis using primers anchored in *PIF5* and *PA03* (and thus spanning the intergenic region) detected the formation of chimeric RNAs specifically in *fpa* mutants (Figure 5B–C). RNA gel blot analysis confirmed this, with a probe to the 5′ end of *PIF5* revealing 60% read-through into RNAs of increased size relative to *PIF5* (Figure 5D). Three major hybridising signals specific to *fpa* mutants were detected using probes spanning the intergenic region, the *PA03* 3′UTR and the different exons and UTRs of *PIF5* (Figure 5D–E). A combination of RT-PCR, 5′ rapid amplification of cDNA ends (RACE), RNA gel blot and cloning approaches identified two of these (α and β) as chimeric RNAs that differ as a result of a cryptic splicing event (Figure 5E–G), while the third comprised two similar sized RNAs with 5′ ends mapping to either the 3′UTR of *PIF5* (γ) or the intergenic sequence (γ′) (Figure 5E–G). The differential sensitivities of these RNAs to tobacco acid pyrophosphatase (TAP) suggest that γ is capped, but γ′ is not (Figure 5F). None of the chimeric RNAs altered the deduced *PIF5* open reading frame, but they did effectively extend the 3′UTR from 211 nt to 2,720 nt in α and to 1,680 nt in β chimeric transcripts. In contrast, native *PA03* expression is undetectable in *fpa* mutants (Figure 5D). The 5′ end of γ RNAs aligned to the *PIF5* 3′UTR (Figure 5F,G) but, as judged by 5′RACE, was distinct from all of the 3′ ends that mapped to the *PIF5* 3′UTR (Figure 5G). This suggests that γ RNAs did not result from cleavage of chimeric RNAs followed by capping. Instead, we found differences between WT and *fpa* mutants in H3K4me3, a chromatin modification associated with transcription start sites [34], across *PIF5* and *PA03*, with a decrease in H3K4me3 at the 5′ end of *PA03* and an additional H3K4me3 peak detected at the 3′ end of *PIF5* in *fpa-8* (Figure 6A). These data are consistent with a shift in the *PA03* transcription start site accompanying the chimeric RNAs detected here in *fpa* mutants.

In addition to being detected in single *fpa-7* and *fpa-8* mutant alleles, chimeric *PIF5–PA03* RNAs were detected in an early-flowering *flc-3 fpa-7* double mutant but were generally not found in other late-flowering mutants (Figure S7A–C), revealing that they result from a specific lack of FPA and not indirectly from late flowering. We did detect low levels of chimeric RNAs in late-flowering *pcfs4* mutants (Figure S7C) in which a protein related to the core cleavage, polyadenylation and termination factor Pcf11 is disrupted [17]. We investigated whether FPA may mediate this effect indirectly by determining whether splicing of *PIF5* pre-mRNA is perturbed, since splicing is intimately connected to RNA 3′ end formation [1]. However, we found no evidence for changes in either the fidelity or efficiency of *PIF5* pre-mRNA splicing in *fpa* mutants (Figure S7D–F). We also investigated whether FPA affects the turnover of chimeric RNAs, since they comprise long 3′UTRs with multiple introns downstream of in-frame stop codons that might normally be degraded by nonsense-mediated RNA decay (NMD) [35]. However, we found no evidence of stabilised read-through RNAs in mutant backgrounds defective in the NMD factor UPF1 [36] (Figure 6B–C), and indeed found that chimeric RNAs appear to escape NMD (Figure 6C). *PIF5* is one of a family of critical growth regulators in *A. thaliana* and is closely related to *PIF4*, with which it shares some functions [37]. *PIF4* and *PIF5* may have arisen from a duplication event since both have related downstream polyamine oxidase loci (*PIF4-PA02* and *PIF5-PA03*) that have conserved peptide open reading frames in their 5′UTRs (*CPuORF17* and *CPuORF18*, respectively). Despite these similarities, chimeric RNAs were found at *PIF5*, but not *PIF4* (Figure S7G) in *fpa* mutants, and not *dcl1* mutants (Figure S7H) underscoring the specificity of FPA-mediated effects on chimeric RNA formation.

Chimeric RNA formation was detected at four other tandem gene pairs (Figures S8–S11) following the development of an algorithm based on reciprocal DRS read abundance at tandem protein-coding genes (Tables S8,S9). In each case, the resulting chimeric RNA encodes the open reading frame for the same upstream gene combined with a different effective 3′UTR (Figure S8C). Remarkably, one chimeric RNA isoform appears to result from a splicing event that excised almost the entire coding sequence of the downstream At1g02470 gene from the chimeric transcript. In all cases, while there was evidence of chimeric RNA in WT, the level of chimeric RNAs was increased in *fpa* mutants. Although our algorithm probably underestimates the number of chimeric RNAs formed in *fpa* mutants because it only considers neighbouring protein-coding genes, it is clear that FPA affects specific chimeric RNA formation at a limited number of sites in the genome.

### Defective termination in *fpa* mutants can occur at the same loci in *dcl* mutants

**Figure 7:**
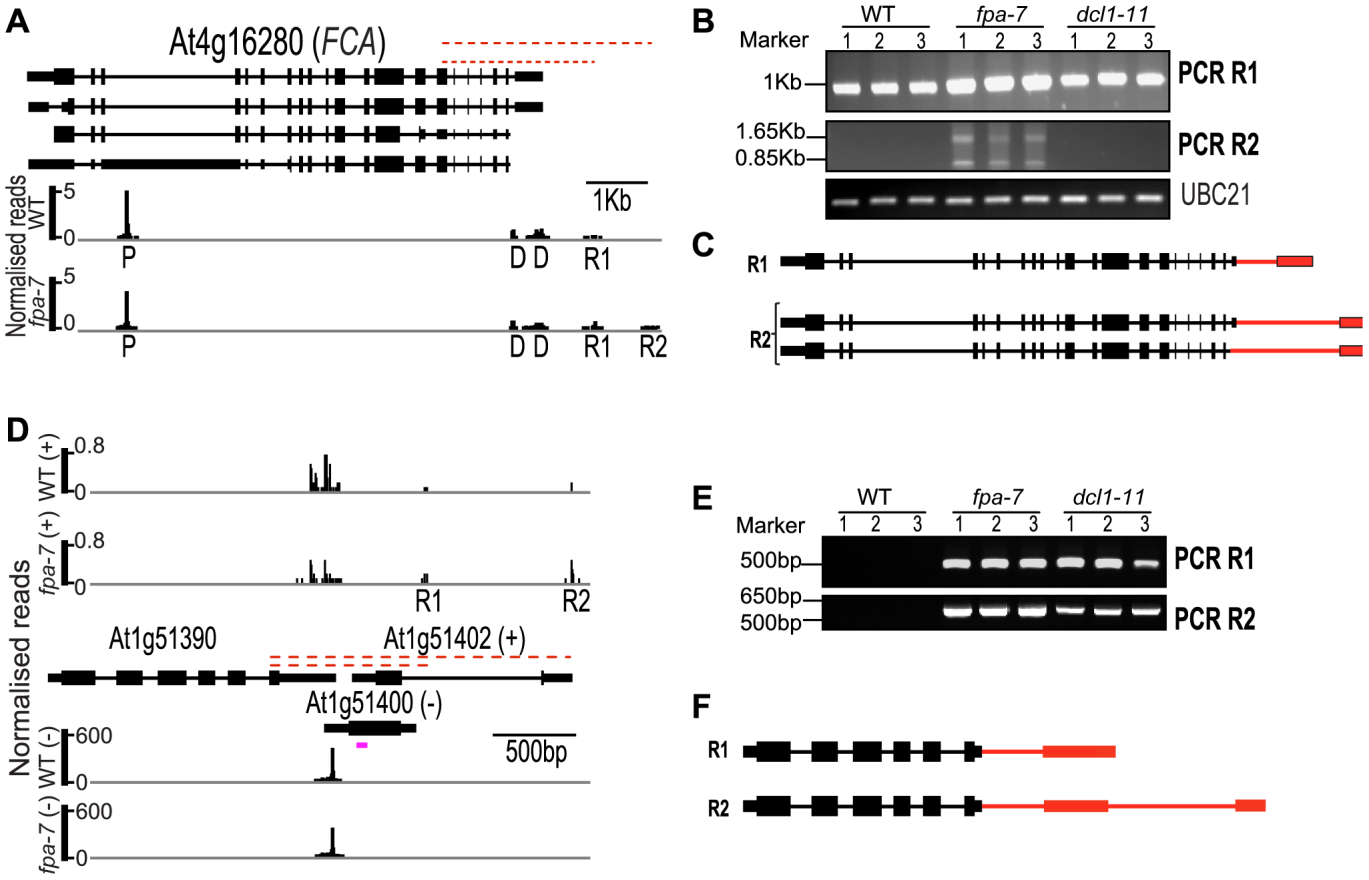
Characterisation of intergenic read-through RNAs in *fpa* and *dc11* mutants. (*A–C*) Characterisation of *FCA* intergenic read-through RNAs. (*A*) Normalised reads mapping to the locus encoding *FCA*. (*B*) Identification of R1 and R2 contiguous RNAs. Three biological replicates (1, 2 and 3) were used for each genotype: wild-type (WT), *fpa*-7 and *dc11–11*. (*C*) Description of the R1 and R2 read-through transcripts. (*D–F*) Characterisation of At1g51390 read-through RNAs. (*D*) Normalised reads mapping to At1g51390–At1g51402–At1g51400. (*E*) Identification of R1 and R2 contiguous RNAs. Three biological replicates (1, 2 and 3) were used for each genotype: WT, *fpa*-7 and *dc11–11*. (*F*) Description of the R1 and R2 read-through transcripts. The red dashed lines indicate the regions amplified by RT-PCR on reverse-transcribed poly(A)+ RNAs. Red narrower rectangles represent 3′UTR parts specific to the read-through transcript and red lines the 3′UTR introns. RT-PCR products were separated on agarose gels and stained with ethidium bromide. The purple line indicates the location and strand detected by the probe, which was used previously [24]. The image of normalised read alignments was made using the Integrated Genome Browser [55] and corresponds to combined reads from the three sequenced biological replicates for each genotype. Exons are denoted by coloured rectangles, UTRs by adjoining narrower rectangles and introns by lines.

Read-through was detected at the 3′ end of the flowering regulator *FCA* (At4g16280) in *fpa* mutants. Failsafe termination at this site was recently shown to depend upon DCL4 [38]. *FCA* read-through RNAs similar to those previously found in *dc14* mutants were detected here in *fpa* mutants (Figure 7A–C), revealing that, even in the presence of DCL4, failsafe termination fails in the absence of FPA. The expression of DCL4 itself is unaffected by FPA (Table S2).

We next asked whether this connection between FPA and DCL proteins extends to other loci. DRS indicated that *fpa* read-through RNAs are found at genomic regions where DCL-dependent small RNAs, described as natural antisense transcript siRNAs (nat-siRNAs), are also found [24]. We first validated the existence of these read-through RNAs in *fpa* mutants, using RT-PCR to reveal read-through at the 3′ end of At1g51390, for example (Figure 7D). A DCL1-dependent nat-siRNA1g51400 has recently been mapped to this genomic region [24]. Using RT-PCR analysis of poly(A)+ RNA, we identified similar read-through RNAs in *dc11–11* downstream of At1g51390 (Figure 7D–F). This finding is consistent with DCL1 ultimately controlling transcription termination. Expression of the annotated downstream gene At1g51402 has previously been reported to increase in *dc11* mutants, which has been taken as evidence of nat-siRNA-mediated gene silencing [24]. However, our findings suggest that this detectable increase in At1g51402 expression can be explained instead by read-through from the upstream gene. We also detected differences in 3′ end formation at other loci in *fpa* and *dc11* mutants, although read-through at *FCA* was unaffected by DCL1 (Figure 7B). We therefore conclude that FPA and DCL proteins control termination at overlapping sets of target genes.

## Discussion

The impact of alternative polyadenylation and transcription termination is underexplored, but widespread changes in poly(A) site choice reveal it to be an important level at which gene expression can be regulated. Here we used DRS to assess the consequences of disrupting regulated 3′ end formation dependent on the spen family protein FPA.

The function of FPA in flowering ultimately depends on its control of the floral repressor *FLC* [15]. The recurrent identification of late-flowering mutants with elevated *FLC* expression, in which other factors that mediate RNA cleavage and polyadenylation are also disrupted, suggests a critical role for RNA 3′ end formation in *FLC* regulation [16]–[18]. However, 3′ end formation of *FLC* mRNA itself appears to be largely unaffected by these mutations. Instead, attention has focused on a potential role for asRNA processing in *FLC* regulation because polyadenylated asRNA expression at the *FLC* locus is also changed in these mutants [14], [18], [39]. Importantly, it is unclear whether these asRNAs are the cause of, the consequence of or simply coincide with *FLC* regulation. For example, the positive correlation of sense and asRNA expression detected here may be a consequence of gene-looping events that juxtapose promoters and terminators that are each characterised by nucleosome-free regions [40]. The embedded nature of these asRNAs within the *FLC* locus makes experimental separation of these events particularly challenging. DRS sheds new light on these complex issues because it can unequivocally score the strand of origin and simultaneously define and quantify multiple sites of RNA 3′ end formation [22], [23]. DRS analysis provided little evidence of altered asRNA proximal site cleavage between WT and *fpa* mutants, while increased levels of asRNAs that read-through to cleavage sites antisense to the *FLC* promoter were clearly detected in *fpa* mutants. These findings are not easily reconciled with a model that suggests proximal processing of asRNA triggers *FLC* chromatin silencing [18]. Clearly, much remains to be explained about *FLC* regulation and, while the DRS data might restrict some models of interpretation, greater sequencing depth and characterisation of further RNA species are also required. For example, it is unclear whether RNAs engaged in R-loops at this locus (within a region that overlaps the proximally polyadenylated *FLC* asRNAs) [41] would be detected by DRS.

A second proposed function for FPA is in RNA-mediated chromatin silencing [21]. Our DRS data, together with recently reported DNA methylation data [27], show that FPA does not play a widespread role in RNA-mediated chromatin silencing at RdDM sites. This clarification is important because a perceived role in RNA silencing has influenced interpretations of how FPA might function. Our data do not explain why FPA was identified in a mutant screen for factors required for RNA silencing [21]. We could show that a novel asRNA, which may be generated by defective termination in *fpa* mutants, was not obligatorily associated with reduced sense strand expression, but we also identified cases in which increased cleavage and polyadenylation antisense to promoter regions in *fpa-7* mutants was associated with either increased or reduced sense strand expression. Therefore, one explanation for the identification of FPA as a factor required for RNA silencing may be that defective 3′ end formation, arising either within the transgenes used for the screen or at endogenous genes close to the site of transgene insertion, affects the efficacy of the silencing trigger from the transgene itself [21]. We did identify misregulation of *psORF*, a recently acquired pseudogene [28], [29], in one *fpa* mutant allele. The silencing of such recently duplicated sequences in different backgrounds is poorly studied, but recent work suggests that epialleles may exist in either different accessions or different generations. In other words, *de novo* originated genes might be prone to epigenetic variation in the early stages of their formation [30]. Since we only detected misregulation of *psORF* in *fpa-7* and not in *fpa-8*, and also did not detect misregulation of asRNAs at the newly acquired At1TE93275 transposable element in this *fpa-7* dataset, it is unclear whether FPA is genuinely involved in the control of such newly arisen sequences.

Besides addressing specific biological roles of FPA in *A. thaliana*, we also asked whether this analysis could reveal generic consequences of defective RNA 3′ end formation. We detected changes in intronic cleavage sites, intergenic read-through and chimeric RNA formation, thus defining a range of events that inform future analyses of the impact of such regulators in other species. These findings also have broader implications because they suggest how 3′ end formation might affect the evolution of gene structure and gene order through regulated 3′UTR sequences, new exon combinations or *de novo* evolution of new genes. We (and others [31]) discovered that intergenic read-through to new poly(A) sites in *fpa* mutants is often associated with cryptic splicing events, consequently generating mRNAs with a diversity of potential 3′UTRs. Furthermore, in some cases read-through was associated with cryptic splicing events that disrupted upstream protein-coding exons, resulting in an impact on protein function that may be more immediately obvious. The strong intergenic poly(A) signals (activated here in *fpa* mutants) may derive from genuine alternative polyadenylation, unannotated genes or transposons or may instead reflect the evolution of high quality intergenic poly(A) sites to trap runaway read-through RNAs. Such read-through to intergenic poly(A) signals may contribute to the *de novo* evolution of new or orphan genes from non-coding genomic regions [42].

One strikingly clear shift in poly(A) site choice was detected at *IBM1*. Alternative polyadenylation of *IBM1* pre-mRNA controls the activity of the histone demethylase encoded by this gene [33]. *IBM1* poly(A) site choice is also dependent on DNA methylation: the *IBM1* intronic DNA sequence downstream of the proximal poly(A) site is heavily methylated and alternative polyadenylation is profoundly altered in *met1* and *cmt3* mutants [33]. Relatively little is known about the impact of DNA methylation and intragenic heterochromatin on co-transcriptional pre-mRNA processing, but alternative splicing was recently shown to be affected by DNA methylation [43]. In addition, alternative polyadenylation of the imprinted H13 locus in mice involves interplay between two poly(A) sites and DNA methylation at an internal promoter that separates them [44]. How directly involved DNA methylation and FPA are in poly(A) site choice at *IBM1* still needs to be established. However, the opposing alternative polyadenylation phenotypes of *met1* and *cmt3*, on the one hand, and *fpa* mutants, on the other, suggest that *A. thaliana IBM1* could be a genetically tractable model system to dissect the interplay between DNA methylation, intragenic heterochromatin and poly(A) site selection. It will be interesting to determine whether other FPA-dependent alternative polyadenylation events are also related to DNA methylation. It may be relevant that methyl-CpG binding domain protein 9 (MDB9) binds to regions of *FLC* chromatin coincident with the sites of alternative polyadenylation of *FLC* asRNAs and influences *FLC* expression and DNA methylation [45].

A striking consequence of read-through we discovered in *fpa* mutants was the formation of chimeric RNAs. Conceptually related chimeric RNAs have previously been discovered [8]–[10], but no specific *trans*-acting factor mediating their formation has been found. Therefore, one of the advances we make here is to reveal that a consequence of losing regulated 3′ end formation can be the transcription of specific chimeric RNAs. We do not yet know whether FPA-dependent chimeric RNAs are regulated *in vivo* or are simply the consequence of loss-of-function in *fpa* mutants. Regardless, chimeric RNAs have been overlooked in genome-wide studies of 3′ end formation until now [46]–[48]. However, the formation of chimeric RNAs may account for phenotypes resulting from defects in mediators of this process or be a consequence of global changes in poly(A) site choice that distinguish quiescent from proliferating cells [49], [50]. Since chimeric RNAs bring together different exon combinations, they have the potential to generate novel biological functions. Consistent with this idea, specific chimeric RNAs are conserved in different vertebrates [10]. An additional function or consequence of chimeric RNA formation that we detected here may be transient silencing of downstream genes by interference with transcription [51] or pre-mRNA processing. By discovering a *trans*-acting factor that affects specific chimeric RNAs, it should now also become possible to design switches that reversibly convert two genes into one in a controllable way and that may have biotechnological applications.

Although it is clear that chimeric RNAs were formed at only a limited number of sites in *fpa* mutants, it is likely that our algorithm identifying reciprocal changes in expression at tandem protein-coding genes underestimated the number of potential chimeric RNAs. For example, Pol II read-through could result in complex transcripts comprised of more than two genes (including non-protein-coding RNAs), which terminate at a site other than the 3′ end of an annotated protein-coding gene. Indeed, a transcript previously detected in *fpa fca* double mutants [31], which can also be described as a chimeric RNA, results from read-through at the 3′ end of At1g55805 into At1g55800 and is cleaved within a downstream intergenic space.

A clear molecular phenotype associated with loss-of-function *fpa* mutants is the stable accumulation of RNAs that are cleaved and polyadenylated in intergenic regions, revealing that FPA ultimately promotes transcription termination. Our analysis of these read-through events led us to make the unexpected discovery that FPA and DCL proteins share termination targets. DCL4, an RNase III-like protein previously established to function in processing small RNAs, was recently also shown to function in transcription termination at the *FCA* locus [38]. One interpretation of these data is that DCL4 mediates ‘failsafe’ termination by cleaving RNA downstream of the poly(A) site(s) at potential CoTC sites and thus facilitates access of the 5′–3′ exonuclease that disrupts the transcribing Pol II. We discovered the same read-through events at the *FCA* locus in *fpa* mutants, revealing that termination at this locus requires not only DCL4 but also FPA. Since FPA does not affect *DCL4* expression, this suggests that termination at certain loci requires multiple, specific *trans*-acting factors in addition to the torpedo exonuclease and the cleavage and polyadenylation machinery [3], [52], [53]. We discovered that the connection between FPA, DCL proteins and termination was not limited to DCL4, since DCL1 may ultimately affect termination at some loci that are also regulated by FPA. Notably, we detected read-through downstream of genes with validated DCL1-dependent antisense nat-siRNAs [24]. DCL proteins may directly cleave nascent RNA to mediate termination, as recently suggested [38] (with small RNAs such as nat-siRNAs simply being the by-products of this cleavage), or may cleave antisense RNAs and thus generate siRNAs that guide subsequent nascent sense strand cleavage and Pol II termination by Argonaute proteins. Notably, read-through at the *FCA* locus in *dc14* mutants also occurs antisense to an overlapping gene, At4g16270 [38]. Here we clarify the suggestion that nat-siRNAs function in *trans* to silence At1g51402 gene expression [24] by revealing that defective termination at the upstream gene in *dc11* mutants can explain these findings. This set of discoveries raises the general question of whether some aspects of *dcl* mutant phenotypes are a direct consequence of defects in Pol II transcription termination.

By focusing our analysis on polyadenylated RNA 3′ ends, we set out to investigate the generic consequences of disrupting a regulator of alternative polyadenylation. We have documented examples of such alterations here, but it will now be interesting to study the mechanism by which FPA mediates this control. Crucial to this is the identification of the immediate, not simply ultimate, targets of FPA function. Furthermore, sequencing RNA from individual cells of different tissues and across circadian rhythms of expression is likely to be required for the comprehensive identification of transcripts affected by FPA.

We recently proposed that the 3′ ends of thousands of *A. thaliana* genes require re-annotation [22]. The more in-depth DRS data we present here reinforces this finding. Although we developed an algorithm designed to assign DRS reads to incompletely annotated gene models, this automated approach used in isolation is not meant to be definitive [22]. Ultimately, the analysis of gene expression would benefit from the development of a technology that directly sequences entire RNA molecules at depth. In the current absence of such a technique, it is likely that accurately revising *A. thaliana* genome annotation will require diverse datasets that include DRS and, for example, RNA-seq and chromatin modification data. The meta-analysis of data from these different technologies nevertheless requires appropriate alignment software specific for each sequencing technology. Single molecule sequencing is more susceptible to mismatches and deletions and so requires indel-aware alignment software. A recent analysis of DRS data is fundamentally flawed because it used a version of the Bowtie alignment software with parameters that are wholly inappropriate for DRS data analysis [54]. Although global analyses of pre-mRNA processing require an improved understanding and annotation of the *A. thaliana* transcriptome, it is likely that *de novo* annotation of control transcriptomes prepared in parallel, rather than improved genome annotation alone, will be required to comprehensively analyse changes in the sequences and levels of RNA molecules resulting from altered RNA processing.

## Materials and Methods

### Plant material and growth conditions

The T-DNA insertion line *fpa-7* (SALK_021959) and the *flc-3* mutant were provided by R. Amasino (Madison). The *fpa-8* mutant (induced by EMS and containing a point mutation leading to a premature stop codon) and *fy-2* were provided by C. Dean (John Innes Centre), and *fve-3* was provided by J. Martínez-Zapater (Madrid). The WT strain Col-0 and the T-DNA insertion lines *upf1–5* (SALK_112922), *sr45*–1 (SALK_004132) and *flk-1* (SALK_007750), as well as *ld-1*, were obtained from NASC (UK). *fld-3* and *ref6–3* were provided by S. Michaels (Indiana University) and Y.S. Noh (Seoul National University), respectively. *A. thaliana* WT Col-0, *fpa-7* and *fpa-8* mutant seeds were sown in MS10 plates, stratified for 2 days at 4°C and germinated in a controlled environment at a constant temperature of 24°C under 16 h light/8 h dark conditions. Seedlings were harvested 14 days after transfer to 24°C.

### RNA procedures

Samples were prepared for DRS as described previously [22] and RNA gel blot analyses were carried out as described [14]. For RT-PCR and RT-qPCR, RNA was isolated using TRI Reagent (Sigma-Aldrich) followed by DNase I treatment (Invitrogen), and reverse transcription was primed with oligo(dT)15 using M-MLV reverse transcriptase (Promega). RT-qPCR was carried out as previously described [14]. For validation of read-through transcripts, fragments were cloned into the pGEM-T Easy (Promega) vector and then sequenced. 5′RACE was performed on 250 ng of poly(A)+ RNA from WT and *fpa-8* backgrounds using the FirstChoice RLM-RACE Kit (Ambion), with or without TAP treatment, according to the manufacturer's instructions. Fragments obtained by 5′RACE were cloned into the pGEM-T Easy (Promega) vector and then sequenced.

### Aligning and filtering procedures for DRS data

Sequencing datasets described in this study have been deposited at the European Nucleotide Archive (ENA): Study, PRJEB3993; accession no, ERP003245. Raw DRS sequences were aligned using open source HeliSphere software (version 1.1.498.63, available free from http://sourceforge.net/projects/openhelisphere/files/helisphere/).

The indexDPgenomic aligner was run with the following parameters: seed_size = 18; num_errors = 1; weight = 16; best_only = 1; max_hit_duplication = 25; percent_error = 0.2; read_step = 4; min_norm_score = 4.2; and strands = both. We discarded globally non-unique alignments and selected one alignment randomly if there were several non-unique local alignments mapped to a genetic region. Reads with more than four indels were deleted, and read alignments were refined using an iterative multiple alignment procedure. DRS reads containing low complexity genomic regions, identified by DustMasker from the Blast+ 2.2.24 package, were discarded, as previously described [22].

### Data normalisation

In order to compare WT and *fpa* mutant data, we used read-per-million (RPM) normalisation, i.e. each DRS read was assigned a weight in such a way that the sum of all weights in a sample condition was equal to 1×10^6^. We called the weights ‘normalised reads’. All images of normalised read alignments were made using the Integrated Genome Browser [55] and correspond to combined reads from the three sequenced biological replicates for each genotype.

### Algorithm for predicted re-annotation of coding genes

An algorithm that accommodates intergenic DRS reads in automated gene re-annotation was carried out as previously described [22], but based on the WT data described in this study.

### Differential expression analyses

The DESeq (version 1.8.2) package [25] was used to search for differentially expressed (DE) genes and poly(A) peaks. DESeq estimates the variance of expression levels for a set of genomic features (genes, intergenic regions or poly(A) peaks) based on read count data within the features in several biological replicates from two different genotypes. This package then calculates *P* values for the features to be non-DE based on the hypothesis that replicated expression levels of the features are distributed according to negative binomial distribution. These *P* values were adjusted using Benjamini-Hochberg multiple testing corrections [56]. We prepared raw un-normalised DRS read counts for all replicates of the two genotypes. In order to curb uncertainty due to low read counts, we set a minimal read count per replicate of 15 raw reads for protein-coding genes and transposons, 11 raw reads for intergenic regions, and 8 raw reads for poly(A) peaks. Reads with counts lower than the limit in different replicates were excluded from the DE analyses. These cutoffs were established by maximising the number of DE features identified between WT and *fpa-7*.

### Differentially expressed coding gene analysis

Counts of DRS reads mapping to re-annotated protein-coding genes were prepared for three replicates of each genotype (WT and *fpa*). A total of 18,406 protein-coding genes were identified. Once the requirement of a minimum raw read count of 15 was applied to all replicates, 15,081 protein-coding genes remained for the DE analysis. Table S2 summarises normalised mean expression values for both genotypes, as well as fold change and *P* values calculated by DESeq for each coding gene with a *P* value of <0.01 to be considered as differentially expressed. Table S3 shows the same data for transposons and transposable gene elements.

### Analysis of antisense RNAs at differentially expressed coding gene promoters

We defined promoter regions as the 2 Kb regions upstream of the transcription start site of protein-coding genes. The analysis was based on prepared poly(A) peaks for intergenic read-through analysis and our list of DE coding genes (Table S2). *P* values between WT and *fpa* were calculated for the regions antisense to DE gene promoter, with at least 11 raw reads in a replicate. So-called promoter regions with *P* values<0.05 in the *fpa* mutants were classified as DE genes with antisense transcripts that overlap with their promoters.

### Putative chimeric RNA analysis

Uniquely aligned and filtered reads and re-annotated coding genes were used for this analysis. We prepared a list of neighbouring genes with normalised read counts for every gene for both genotypes (WT and *fpa*). We selected gene pairs with expression levels higher than 0.5 RPM (this corresponds to roughly 11–12 raw reads within a gene) for both genes in the pair and for both genotypes. Pairs with a DESeq *P* value of <0.01 for which the upstream gene was down-regulated and the downstream gene was up-regulated in the *fpa* mutant were considered as candidates for chimeric RNA formation.

### Gaussian smoothing of DRS data and peak-finding algorithm

We prepared a dataset of poly(A) sites, corresponding to the 3′ ends of aligned and filtered DRS reads, by applying smoothing and peak-finding algorithms as previously described [22].

### Analysis of differentially expressed intergenic read-throughs

We defined intergenic regions as regions between protein-coding genes and excluded genomic regions of transposable gene elements, pseudogenes and non-coding genes. The analysis was based on uniquely aligned, filtered and smoothed poly(A) sites and our re-annotation of coding genes. We separately prepared poly(A) peaks for each replicate, clustered poly(A) peaks within a 4-bp window and selected poly(A) peaks with at least 0.5 RPM expression. We removed poly(A) peaks within protein-coding genes, transposable gene elements, pseudogenes and non-coding genes. Since we did not re-annotate the transposable gene elements, pseudogenes and non-coding genes, we also excluded poly(A) peaks within 50 bp downstream of the 3′ ends of these genomic features. We assigned two parameters to every intergenic region: the total sum of poly(A) peaks within the region (and for each replicate); and the position of the centroid of the poly(A) peaks in a region as P_centroid_ = ΣE_i_P_i_/ΣE_i_, where E_i_ is the expression level of i^th^ poly(A) peak within each region and P_i_ is the genomic coordinate of the peak. *P* values between WT and *fpa* were calculated for intergenic regions with at least 11 raw reads in a replicate. At the final step, intergenic regions with *P* values<0.05 and centroid positions not within 30 bp downstream of the coding gene 3′ end in the *fpa* mutants were classified as DE. The 30-bp offset corresponds to the median length of DRS reads; by applying this additional filter we therefore excluded situations where intergenic reads were mainly grouped near the 3′ end of the upstream gene and hence may belong to the gene.

### Analysis of differentially expressed poly(A) peaks

In this analysis, we compared expression levels of individual poly(A) peaks in the two genotypes rather than the total reads aligned to a gene. The comparison of peaks is complicated by over-calling peaks (i.e. peaks being identified where there should be no peak) and the fact that the centre of biologically equivalent peaks may be called in slightly different positions in different genotypes as a consequence of the varied read depth and the peak-calling algorithm. Figure S12 summarises the process we developed to overcome these issues and systematically identify equivalent peaks between WT and *fpa-7*.

Having identified peak pairs, we applied DESeq to all pairs with at least 8 raw reads in a replicate. This gave 86,699 poly(A) peak pairs. Peak pairs with a *P* value of <0.01 were considered to be DE in the *fpa-7* mutant. These comprised 6% of the selected poly(A) peaks: 2,538 down-regulated and 2,671 up-regulated. Due to the data partition between two neighbouring peaks in one genotype and/or incorrect peak matching, our algorithms can produce ‘false positives’. For example, in Figure S13, if we examine the most highly expressed poly(A) peak in the gene, we see there is another poly(A) peak downstream of the peak in WT and nothing in *fpa-7*. The peak-finding algorithm incorrectly combined poly(A) sites belonging to the bump on the right-hand slope of the peak to the most highly expressed poly(A) peak in *fpa-7* and created a new peak in WT. Since this WT peak was matched to 0 in *fpa-7*, it was wrongly classified as DE by DESeq. We therefore designed an algorithm to identify and exclude these ‘false positive’ peaks: if a poly(A) peak was up-regulated in one genotype, we looked for poly(A) peaks within an 8-bp window in the other genotype; if we found poly(A) peaks in the genotype that were not associated with the DE poly(A) peak and the summed expression of the peaks was significantly different (20% higher than the associated poly(A) peak only), then we defined this DE poly(A) peak as a ‘false positive’. This led to 37% of down-regulated and 32% of up-regulated DE peaks being excluded from further analysis. We also applied this method to select intronic cleavage sites that were differentially used in *fpa-7* compared to WT. We then only considered peaks that comprised >10% of expression at a particular gene and manually excluded sites that mapped to 3′UTR introns.

### 3′RACE

3′RACE was performed using the FirstChoice RLM-RACE Kit Protocol (Ambion) according to the manufacturer's instructions. The reaction was started with 250 ng poly(A)+ RNA. Multiple PCR products were purified, cloned into the pGEM-T Easy vector (Promega) and sequenced.

### Chromatin ImmunoPrecipitation (ChIP) analysis

ChIP was performed as previously described [57]. Anti-H3K4me3 monoclonal antibodies were obtained from Diagenode (MAb-152–050).

### Primers used in this study

All primers used in this study are listed in Table S10.

## Supporting Information

Figure S1 Changes between WT and *fpa* mutant backgrounds in polyadenylated RNAs transcribed in the vicinity of the *FLC* locus. (A) DRS Reads mapping to *FLC* locus. Normalised reads are presented for wild-type (WT) and *fpa A. thaliana*. Genes are orientated 5′–3′; exons are denoted by rectangles, UTRs by adjoining narrower rectangles and introns by lines. The image of normalised read alignments was made using the Integrated Genome Browser [55] and corresponds to combined reads from the three sequenced biological replicates for each genotype. Location of RT-qPCR primers [18] are displayed (promoter proximal lncasRNAs, red line; distal polyadenylated lncasRNAs, green line). Identification of proximally polyadenylated *FLC* lncasRNAs in *fpa-7* and WT was based on a low number of reads at surrounding sites, while the distal ‘D’ cleavage sites were clearly defined. In contrast to our findings, reduced levels of proximally polyadenylated lncasRNAs in *fpa* mutants relative to WT have been reported [18]. Differences between the two studies may be clarified by the DRS data in two ways: first, RT-qPCR primers used previously [18] target a single, relatively infrequently used cleavage site (see Figure S1B) and may therefore fail to accurately quantify the expression of polyadenylated read-through mRNAs; and second, Liu *et al.* expressed proximally polyadenylated transcripts as a fraction of the total lncasRNA transcripts [18]. Since more lncasRNA read-through occurs in *fpa* mutants, the proportion of total lncasRNA polyadenylated at the proximal site is indeed reduced, but the DRS data indicate that there is no clear change to proximal cleavage site usage. (*B*) Enlarged image of normalised DRS reads that mapped to lncasRNAs cleaved and polyadenylated antisense to the *FLC* promoter. The enlarged region is boxed in Figure S1A. The A- and U-rich motifs associated with the preferred poly(A) site (black triangle) are displayed. Location of the RT-qPCR primer from Liu *et al.* (2010) is shown by a green line and sites selected by this primer are coloured green. The single nucleotide polymorphism (SNP) described by Coustham *et al.* (2012) is also displayed (red triangle). The preferred distal cleavage site has *cis*-elements typical of preferred cleavage sites in *A. thaliana* 3′UTRs [22], including AAUAAA at −19 (with respect to the preferred cleavage site) and a U-rich hexamer UUGUGU at −9. A SNP at position −121 with respect to the *FLC* transcription start site that is associated with changes in vernalisation requirement, flowering time and the abundance of cold-induced distally polyadenylated lncasRNA [26] maps to the U-rich hexamer at −9, upstream of the preferred cleavage site in lncasRNAs.
(PDF)

Figure S2 Differentially expressed transposons between wild-type and *fpa-7*. (*A*) Analysis of DNA methylation pattern in *fpa-7* at At5g35935 from data published previously [27]. According to recently reported DNA methylation data, a reduction in DNA methylation at *psORF* can be observed in *fpa-7* [27]. DRS data showed that silencing of *psORF* is lost in *fpa-7* (see Figure 3D). CG methylation is in green, CHH in red and CHG in blue [27]. (*B*) Analysis of DRS reads at the At1TE93275 locus. Misregulation of antisense RNAs at the newly acquired helitron transposable element At1TE93275 has previously been reported in *fpa* mutants [31]. These antisense RNAs, described in [31], correspond to the previously unannotated (UA) genomic segment UA228 located in an intergenic region. DRS analysis did not reveal an increase in these antisense RNAs in this *fpa-7* dataset. Normalised reads mapping to the different loci are presented for wild-type (WT) and *fpa*. The top panel displays the reads corresponding to the (+) strand while the bottom panel displays the reads corresponding to the (−) strand. Exons are denoted by rectangles, UTRs by adjoining narrower rectangles and introns by lines. Transposable elements are in red and the UA228 segment in green.
(PDF)

Figure S3 FPA-dependent alternative polyadenylation events. (*A*) Location of the poly(A) sites ‘T’ due to the insertion in *fpa-7*. (*B*) Additional cleavage sites found within intron 4 upstream of the *fpa-7* T-DNA insertion. DRS reads that map to *fpa-7* T1 sites are displayed. The positions of 3′RACE products are indicated above the DNA sequence by red triangles (numbers indicate how many 3′RACE clones were obtained). Images of normalised read alignments were made using the Integrated Genome Browser [55] and correspond to combined reads from the three sequenced biological replicates for each genotype. (*C*) Additional cleavage sites found within intron 5 upstream of the *fpa-7* T-DNA insertion. DRS reads that map to *fpa-7* T2 sites are displayed. Positions of 3′RACE products are indicated on the DNA sequence by red triangles (the numbers indicate how many 3′RACE clones were obtained). (*D*) Additional cleavage sites found within intron 5 upstream of the *fpa-7* T-DNA insertion. Positions of 3′RACE products are marked above the DNA sequence by red triangles (the numbers indicate how many 3′RACE clones were obtained), while DRS reads that aligned to the *FPA* T-DNA junction are marked by blue triangles (with the number of raw reads shown above). A black line indicates the T-DNA sequence. (*E*) 3′RACE analysis of the *mom1–3* mutant. Positions of 3′RACE products are marked on the DNA sequence by red triangles (numbers indicate how many 3′RACE clones were obtained). As the T-DNA mutant *mom1–3* also displayed cryptic cleavage sites upstream of its T-DNA insertion, it is possible that premature cleavage and polyadenylation may form part of the mechanism by which T-DNAs disrupt gene function; this raises the general question of how cryptic sites are triggered. (F) Structure of the T-DNA. The left and right borders (‘LB’ and ‘RB’, respectively) are indicated by red boxes. NPTII is the kanamycin resistance gene used for selection.
(PDF)

Figure S4 FPA affects intronic cleavage site selection. (*A*) Predicted IBM1 protein domain organisation encoded by mRNAs cleaved and polyadenylated at promoter proximal and distal poly(A) sites. (*B*) Reads mapping to the locus encoding *IBM1*. Promoter proximal ‘P’ and distal ‘D’ alternative poly(A) sites are indicated. The probe used for RNA gel blot analysis of alternatively polyadenylated transcripts is indicated in red [33]. (*C*) RNA gel blot analysis of *IBM1* alternatively polyadenylated transcripts. *β-TUBULIN* (*β-TUB.*) was used as an internal control. (*D*) Reads mapping to At1g56500. Promoter proximal ‘P’ and distal ‘D’ alternative poly(A) sites are indicated. The probe used for RNA gel blot analysis of alternatively polyadenylated transcripts is indicated by a red line. (*E*) The predicted protein domain organisation encoded by At1g56500 mRNAs cleaved and polyadenylated at promoter proximal and distal poly(A) sites. At1g56500 encodes a putative hydrolase, but RNA cleavage at the proximal site eliminates a sequence encoding the putative TlpA-like TRX domain and three NHL (NCL-1, HT2A and LIN-41) repeats, while the sequence encoding the hydrolase domain (HAD-like) is retained. (*F*) RNA gel blot analysis of At1g56500 alternatively polyadenylated transcripts. *β-TUBULIN* (*β-TUB.*) was used as an internal control. Quantification of the bands revealed that At1g56500 polyadenylation at the distal and proximal sites is 18% and 82%, respectively, in *fpa-7*; 15% and 85% in *fpa-7 flc-3*; and 30% and 70% in wild-type (WT). (G) Reads mapping to At5g35170. Promoter proximal ‘P’ and distal ‘D’ alternative poly(A) sites are indicated. The probe used for RNA gel blot analysis of alternatively polyadenylated transcripts is indicated by a red line. (*H*) The predicted protein domain organisation encoded by At5g35170 mRNAs cleaved and polyadenylated at promoter proximal and distal poly(A) sites. At5g35170 encodes a putative adenylate kinase, but RNA cleavage at the proximal site eliminates a domain of unknown function (DUF1995). (*I*) RNA gel blot analysis of At5g35170 alternatively polyadenylated transcripts. *β-TUBULIN* (*β-TUB.*) was used as an internal control. The asterisk indicates a non-specific hybridisation signal. Quantification of the bands revealed that At5g35170 polyadenylation at the distal and proximal sites is 81% and 19%, respectively, in *fpa-7*; 92% and 8% in WT; 73% and 27% in *fpa-7*; and 75% and 25% *fpa-7 flc-3*. (*J*) Distally polyadenylated transcripts at At5g35170 exhibit various cryptic splicing sites in the region located between exons 7 and 12 (purple box). Details of the region located between exons 7 and 12 are shown for each isoform. Images of normalised read alignments were made using the Integrated Genome Browser [55] and correspond to combined reads from the three sequenced biological replicates for each genotype.
(PDF)

Figure S5 FPA affects intergenic read-through. (*A*) Histogram of the read-through percentage in *fpa-7* for protein-coding genes with differentially expressed downstream intergenic regions. (*B-E*) Characterisation of *MKK5* intergenic read-through RNAs. (*B*) Normalised reads mapping to the *MKK5* locus. The red dashed line indicates the region amplified by RT-PCR. (*C*) RNA gel blot analysis of *MKK5* read-through transcripts. The probe used is indicated by a red solid line in (*B*). (*D*) Transcripts are either cleaved and polyadenylated in the annotated 3′UTR or at the intergenic sites, as determined by sequencing the cloned RT-PCR products. Red rectangles represent the 3′UTR specific to the read-through transcript and red lines represent 3′UTR introns. (*E*) Identification of contiguous RNA from the *MKK5* gene upstream to the intergenic cleavage sites. RT-PCR products were separated on agarose gels and stained with ethidium bromide. Amplification controls for genomic DNA contamination (RT-) are included. Three biological replicates (1, 2 and 3) were used for each genotype: wild-type (WT) and *fpa*-7. (*F–Q*) Selection of tested read-through RNAs. (*F, I, L, O*) Normalised reads mapping to the analysed loci. Red dashed lines indicate the region amplified by RT-PCR. Images of normalised read alignments were made using the Integrated Genome Browser [55] and correspond to combined reads from the three sequenced biological replicates for each genotype. (*G, J, M, P*) Identification of contiguous RNAs from the upstream gene to the intergenic poly(A) sites. RT-PCR products were separated on agarose gels and stained with ethidium bromide. Amplification controls for genomic DNA contamination (RT-) are included. Three biological replicates (1, 2 and 3) were used for each genotype: WT and *fpa*-7. (*H, K, N, Q*) Transcripts are either cleaved and polyadenylated in the annotated 3′UTR or at the intergenic sites, as determined by sequencing the cloned RT-PCR products. Narrower red rectangles represent 3′UTRs specific to the read-through transcripts. (*Q*) Read-through at At5g66450 is accompanied by cryptic splicing that alters the protein-coding sequence.
(PDF)

Figure S6 Schematic representation of a selection of tested read-through RNAs. (*A–F*) Left panels represent normalised reads mapping to the analysed loci. Red dashed lines indicate the regions amplified by RT-PCR. Images of normalised read alignments were made using the Integrated Genome Browser [55] and correspond to combined reads from the three sequenced biological replicates for each genotype. Top right panels show the identification of contiguous RNA from the upstream gene to the intergenic cleavage sites. RT-PCR products were separated on agarose gels and stained with ethidium bromide. Amplification controls for genomic DNA contamination (RT-) are included. Three biological replicates (1, 2 and 3) were used for each genotype: wild-type (WT) and *fpa*-7. Bottom right panels schematically show transcripts either cleaved and polyadenylated in the annotated 3′UTR or at the intergenic sites, as determined by sequencing the cloned RT-PCR products. Narrower red rectangles represent the 3′UTR section specific to the read-through transcript and red lines indicate the 3′UTR introns.
(PDF)

Figure S7 Analysis of the potential causes of *PIF5–PA03* chimeric RNA formation. (*A*) RNA gel blot analysis of *PIF5–PA03* chimeric RNAs in *fpa* and *flc* mutants. P, *PIF5*; C, *PA03* transcripts. *β-TUBULIN* (*β-TUB.*) was used as an internal control. Probes used are shown in Figure 5*B*. (*B,C*) RT-qPCR analysis of *PIF5–PA03* chimeric RNAs in *fpa* and *flc* (*B*) and late-flowering (*C*) mutants. Location of the RT-qPCR amplicon is displayed in (*D*) by a red dotted line and labelled qPCR. *PIF5–PA03* chimeric RNAs were quantified by RT-qPCR analysis of the expression of the intergenic fragment between *PIF5* and *PA03*. Data are the means ± SEM obtained for three independent PCR amplifications of three biological replicates. The y-axis shows the fold change relative to wild-type (WT; set to 1) after normalisation to *UBC21* gene expression. *, *P*<0.05; Student's t-test. (*D*) Structure of the *PIF5–PA03* locus: exons are denoted by yellow rectangles, UTRs by narrower black rectangles and introns by grey lines; regions analysed for splicing efficiency and accuracy are indicated (1, red; 2, green; 3, pink; and 4, blue). The red dashed line indicates the location of the RT-qPCR amplicon. (*E*) Expression levels of *PIF5* spliced RNA for primer sets 1, 2, 3 and 4; their location is displayed in (*D*). Histograms show means ± SEM obtained from three PCR reactions for each of three biological replicates. (*F*) Electropherograms for primer sets 1, 2, 3 and 4. The forward primer in each set was 6-FAM labelled. Numbers on the x-axis represent size markers in nucleotides; numbers on the y-axis represent relative fluorescence ×10^3^, reflecting transcript abundance. Electropherograms are representative of three biological and three PCR replicates. The main splicing product is identified by its size. No significant changes in the ratios of alternatively spliced transcripts were observed between WT and *fpa-8.* (*G*) Normalised reads mapping to loci encoding *PIF4 and PA02*. Images of normalised read alignments on the left panel were made using the Integrated Genome Browser [55] and correspond to combined reads from the three sequenced biological replicates for each genotype. On the right panel, RT-PCR analysis of a contiguous RNA between *PIF4* and *PA02* is displayed but does not reveal formation of chimeric RNAs at this locus in *fpa-7*. The region amplified by RT-PCR is indicated by a red dashed line. RT-PCR products were separated on agarose gels and stained with ethidium bromide. Amplification controls for genomic DNA contamination (RT-) are included. Three biological replicates (1, 2 and 3) were used for each genotype: WT and *fpa*-7. *UBIQUITIN LIGASE 21* (*UBC21*) was used as a control, as well as gDNA for the chimeric RNA analysis. (*H*) RT-PCR analysis of the contiguous RNA between *PIF5* and *PA03* detected in *fpa-7* but not in *dc11–11*. *UBIQUITIN LIGASE 21* (*UBC21*) was used as a control. Three biological replicates (1, 2 and 3) were used for each genotype: WT and *fpa*-7.
(PDF)

Figure S8 Analysis of chimeric RNAs formed between At1g02475 and At1g02470. (*A*) Normalised reads mapping to the At1g02475–At1g02470 loci. Exons are denoted by rectangles, UTRs by adjoining narrower rectangles and introns by lines. Images of normalised read alignments were made using the Integrated Genome Browser [55] and correspond to combined reads from the three sequenced biological replicates for each genotype. *(B)* Chimeric RNAs identified by RT-PCR. The region amplified by RT-PCR is indicated by a red dashed line. RT-PCR products were separated on agarose gels and stained with ethidium bromide. Amplification controls for genomic DNA contamination (RT-) are included. Three biological replicates (1, 2 and 3) were used for each genotype: wild-type (WT) and *fpa*-7. (*C*) Schematic representation of the chimeric RNAs, as determined by sequencing the cloned RT-PCR products. A red line depicts splicing events. (*D*) RT-qPCR analysis of the different RNAs expressed at the At1g02475–At1g02470 loci in *fpa* mutants. Data are the means ± SEM obtained for three independent PCR amplifications on three biological replicates. The y-axis shows the fold change relative to wild-type (set to 1) after normalisation to *UBC21* gene expression.
(PDF)

Figure S9 Analysis of chimeric RNAs formed between At2g32320 and At2g32340. (*A*) Normalised reads mapping to the At2g32320–At2g32340 loci. Exons are denoted by rectangles, UTRs by adjoining narrower rectangles and introns by lines. Images of normalised read alignments were made using the Integrated Genome Browser [55] and correspond to combined reads from the three sequenced biological replicates for each genotype. (*B*) Chimeric RNAs identified by RT-PCR. The region amplified by RT-PCR is indicated by a red dashed line. RT-PCR products were separated on agarose gels and stained with ethidium bromide. Amplification controls for genomic DNA contamination (RT-) are included. Three biological replicates (1, 2 and 3) were used for each genotype: wild-type (WT) and *fpa*-7. (*C*) Schematic representation of the chimeric RNAs, as determined by sequencing the cloned RT-PCR products. A red line depicts splicing events. (*D*) RT-qPCR analysis of the different RNAs expressed at the At2g32320–At2g32340 locus in *fpa* mutants. Data are the means ± SEM obtained for three independent PCR amplifications of three biological replicates. The y-axis shows the fold change relative to WT (set to 1) after normalisation to *UBC21* gene expression. Chimeric RNAs formed between At2g32320 and At2g32340 did not alter the coding potential of the upstream gene but changed the effective 3′UTR.
(PDF)

Figure S10 Analysis of chimeric RNAs formed between At3g06700 and At3g06690. (*A*) Normalised reads mapping to the At3g06700–At3g06690 loci. Exons are denoted by rectangles, UTRs by adjoining narrower rectangles and introns by lines. Images of normalised read alignments were made using the Integrated Genome Browser [55] and correspond to combined reads from the three sequenced biological replicates for each genotype. (*B*) Chimeric RNAs identified by RT-PCR. The region amplified by RT-PCR is indicated by a red dashed line. RT-PCR products were separated on agarose gels and stained with ethidium bromide. Amplification controls for genomic DNA contamination (RT-) are included. Three biological replicates (1, 2 and 3) were used for each genotype: wild-type (WT) and *fpa*-7. (*C*) Schematic representation of the chimeric RNAs, as determined by sequencing the cloned RT-PCR products. A red line depicts splicing events. (*D*) RT-qPCR analysis of the different RNAs expressed at the At3g06700–At3g06690 loci in *fpa* mutants. Data are the means ± SEM obtained for three independent PCR amplifications of three biological replicates. The y-axis shows the fold change relative to WT (set to 1) after normalisation to *UBC21* gene expression. Chimeric RNAs formed between At3g06700 and At3g06690 did not alter the coding potential of the upstream gene but changed the effective 3′UTR.
(PDF)

Figure S11 Analysis of putative chimeric RNAs formed between At1g74880 and At1g74875. (*A*) Normalised reads mapping to the At1g74880–At1g74875 locus. Exons are denoted by rectangles, UTRs by adjoining narrower rectangles and introns by lines. Images of normalised read alignments were made using the Integrated Genome Browser [55] and correspond to combined reads from the three sequenced biological replicates for each genotype. (*B*) Chimeric RNAs were identified by RT-PCR. The region amplified by RT-PCR is indicated by a red dashed line. RT-PCR products were separated on agarose gels and stained with ethidium bromide. Amplification controls for genomic DNA contamination (RT-) are included. (*C*) Schematic representation of the chimeric RNAs, as determined by sequencing of cloned RT-PCR products. A red line depicts splicing events. (*D*) RT-qPCR analysis of the different RNAs expressed at the At1g74880–At1g74875 locus in *fpa* mutants. Data are the means ± SEM obtained for three independent PCR amplifications on three biological replicates. The y-axis shows the fold change relative to wild-type (WT; set to 1) after normalisation to *UBC21* gene expression. The significance of this validated example of chimeric RNA formation (At1g74875 and At1g74880) is less clear because the downstream gene is relatively poorly characterised; it may reflect annotation errors rather than chimeric RNA formation between two well-characterised genes.
(PDF)

Figure S12 Illustration of the peak combination and peak-matching algorithms. (A) Grouping algorithm: (i) stylised smoothed read profile; (ii) peaks called by our algorithm as previously described [1]–[3], [22] (ii) peaks that are close together are often due to errors in peak calling and so are combined as follows. For each peak, a search is made in the 8-bp sequence 3′ of the peak. If a further peak is found, then the two peaks are combined, with a new peak being established at the centroid of the two positions. The value of 8 bp was chosen by experimentation. (B) Peak-matching algorithm: for each peak in wild-type (WT), an equivalent peak in *fpa-7* is identified if it is within +/−5 bp. Peaks A and B were matched. If two peaks are found, then the first is taken as the equivalent peak and the second is assigned to zero. Peaks C1 and D were matched; peak C2 is matched with 0 in WT. Images of normalised read alignments were made using the Integrated Genome Browser [55] and correspond to combined reads from the three sequenced biological replicates for each genotype.
(PDF)

Figure S13 Example of a ‘false positive’ differentially expressed poly(A) peak and matched poly(A) peaks at At2g28000. Top and bottom panels, smoothed raw poly(A) sites mapping to At2g28000; middle panels, poly(A) peaks called from these smoothed poly(A) profiles. All data were subject to read-per-million (RPM) normalisation. Images of normalised read alignments were made using the Integrated Genome Browser [55] and correspond to combined reads from the three sequenced biological replicates for each genotype.
(PDF)

Table S1 Classification of reads mapping to *A. thaliana* genome release, TAIR10.
(XLSX)

Table S2 List of protein-coding genes differentially expressed in *fpa-7*. Normalised reads (reads per million, RPM) are displayed for wild-type (WT) and fpa-7. * When WT read number is ‘0’, fold change is set to ‘1,000,000’.
(XLSX)

Table S3 List of transposons differentially expressed in *fpa-7*. Normalised reads (reads per million, RPM) are displayed. * When wild-type (WT) read number is ‘0’, fold change is set to ‘1,000,000’.
(XLSX)

Table S4 List of Poly(A) peaks differentially expressed in *fpa-7*. Normalised reads (reads per million, RPM) are displayed. * When wild-type (WT) read number is ‘0’, fold change is set to ‘1,000,000’.
(XLSX)

Table S5 List of the intergenic regions differentially expressed in *fpa-7*. Normalised reads (reads per million, RPM) are displayed. * When wild-type (WT) intergenic read number is ‘0’, distance for WT is set to ‘−1’.
(XLSX)

Table S6 List of the intergenic regions with differentially expressed peaks in *fpa-7*. Normalised reads (reads per million, RPM) are displayed. * When the antisense gene is not expressed, N/A is indicated.
(XLSX)

Table S7 List of differentially expressed peaks located in promoters of genes differentially expressed in *fpa-7*. Normalised reads (reads per million, RPM) are displayed. * When wild-type (WT) read number is ‘0’, fold change is set to ‘1,000,000’.
(XLSX)

Table S8 List of putative chimeric RNAs. Normalised reads (reads per million, RPM) are displayed.
(XLSX)

Table S9 List of tested putative chimeric RNAs. Normalised reads (reads per million, RPM) are displayed.
(XLSX)

Table S10 List of primers used in this study.
(XLSX)
