# Supplementary figures and images for: Transcription Termination and Chimeric RNA Formation Controlled by Arabidopsis thaliana FPA
Source: PLoS Genet. 2013 Oct 31;9(10):e1003867. doi: 10.1371/journal.pgen.1003867 (PMC3814327; doi:10.1371/journal.pgen.1003867)

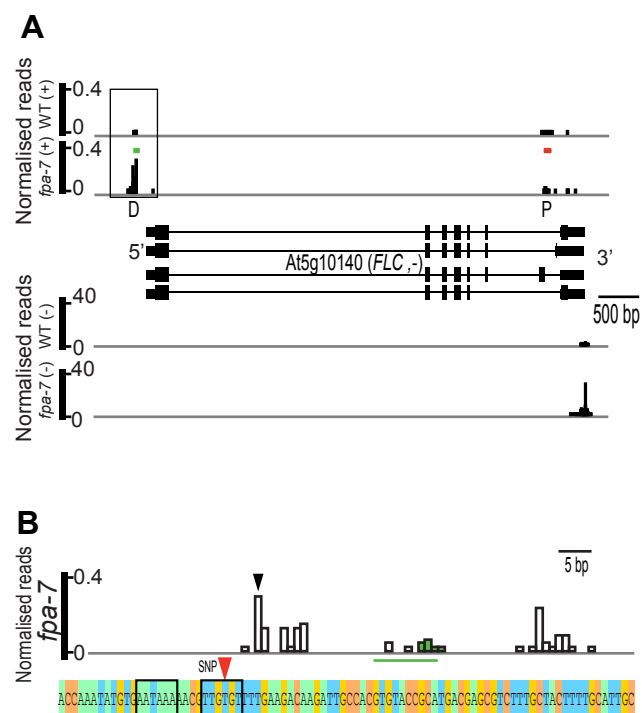

Figure S1. Analysis of DRS reads at *FLC* locus

Supplement: Figure S1 — Changes between WT and fpa mutant backgrounds in polyadenylated RNAs transcribed in the vicinity of the FLC locus. (A) DRS Reads mapping to FLC locus. Normalised reads are presented for wild-type (WT) and fpa A. thaliana. Genes are orientated 5′–3′; exons are denoted by rectangles, UTRs by adjoining narrower rectangles and introns by lines. The image of normalised read alignments was made using the Integrated Genome Browser [55] and corresponds to combined reads from the three sequenced biological replicates for each genotype. Location of RT-qPCR primers [18] are displayed (promoter proximal lncasRNAs, red line; distal polyadenylated lncasRNAs, green line). Identification of proximally polyadenylated FLC lncasRNAs in fpa-7 and WT was based on a low number of reads at surrounding sites, while the distal ‘D’ cleavage sites were clearly defined. In contrast to our findings, reduced levels of proximally polyadenylated lncasRNAs in fpa mutants relative to WT have been reported [18]. Differences between the two studies may be clarified by the DRS data in two ways: first, RT-qPCR primers used previously [18] target a single, relatively infrequently used cleavage site (see Figure S1B) and may therefore fail to accurately quantify the expression of polyadenylated read-through mRNAs; and second, Liu et al. expressed proximally polyadenylated transcripts as a fraction of the total lncasRNA transcripts [18]. Since more lncasRNA read-through occurs in fpa mutants, the proportion of total lncasRNA polyadenylated at the proximal site is indeed reduced, but the DRS data indicate that there is no clear change to proximal cleavage site usage. (B) Enlarged image of normalised DRS reads that mapped to lncasRNAs cleaved and polyadenylated antisense to the FLC promoter. The enlarged region is boxed in Figure S1A. The A- and U-rich motifs associated with the preferred poly(A) site (black triangle) are displayed. Location of the RT-qPCR primer from Liu et al. (2010) is shown by a green lin [file pgen.1003867.s001.pdf]

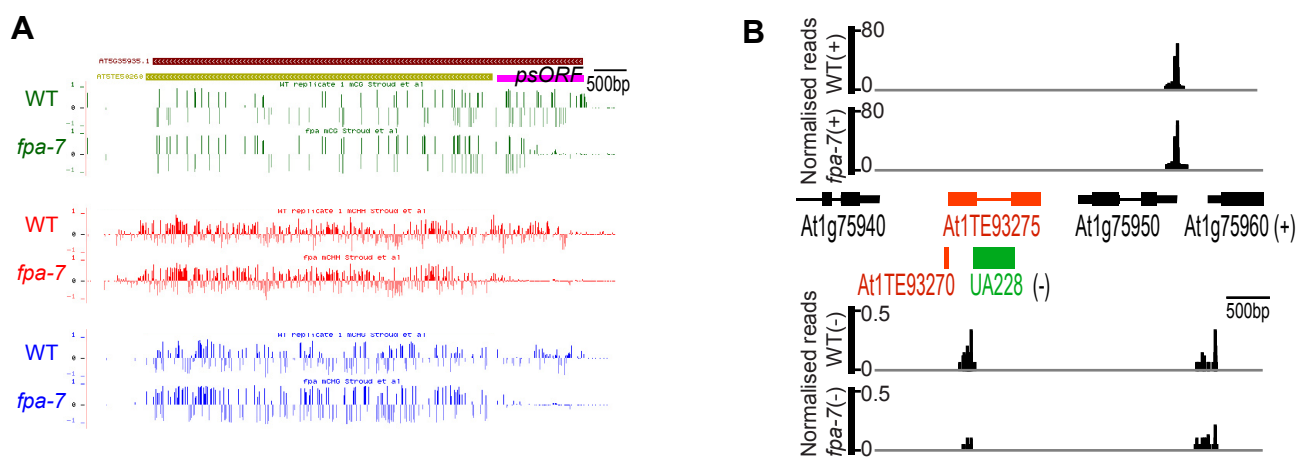

Figure S2. Differentially expressed transposons in WT and *fpa-7*.

Supplement: Figure S2 — Differentially expressed transposons between wild-type and fpa-7. (A) Analysis of DNA methylation pattern in fpa-7 at At5g35935 from data published previously [27]. According to recently reported DNA methylation data, a reduction in DNA methylation at psORF can be observed in fpa-7 [27]. DRS data showed that silencing of psORF is lost in fpa-7 (see Figure 3D). CG methylation is in green, CHH in red and CHG in blue [27]. (B) Analysis of DRS reads at the At1TE93275 locus. Misregulation of antisense RNAs at the newly acquired helitron transposable element At1TE93275 has previously been reported in fpa mutants [31]. These antisense RNAs, described in [31], correspond to the previously unannotated (UA) genomic segment UA228 located in an intergenic region. DRS analysis did not reveal an increase in these antisense RNAs in this fpa-7 dataset. Normalised reads mapping to the different loci are presented for wild-type (WT) and fpa. The top panel displays the reads corresponding to the (+) strand while the bottom panel displays the reads corresponding to the (−) strand. Exons are denoted by rectangles, UTRs by adjoining narrower rectangles and introns by lines. Transposable elements are in red and the UA228 segment in green. (PDF) [file pgen.1003867.s002.pdf]

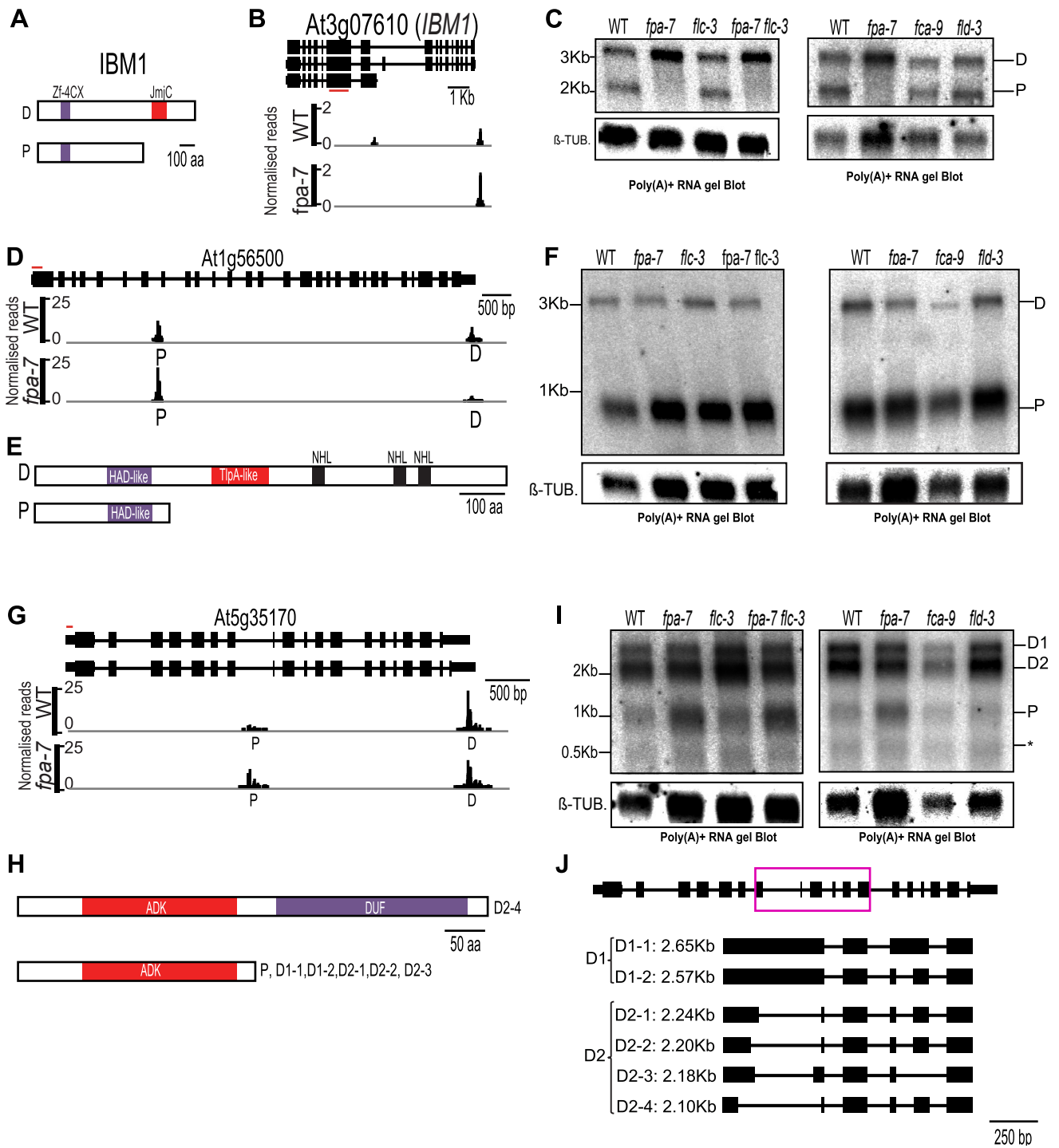

Figure S4. Analysis of alternatively polyadenylated transcripts.

Supplement: Figure S4 — FPA affects intronic cleavage site selection. (A) Predicted IBM1 protein domain organisation encoded by mRNAs cleaved and polyadenylated at promoter proximal and distal poly(A) sites. (B) Reads mapping to the locus encoding IBM1. Promoter proximal ‘P’ and distal ‘D’ alternative poly(A) sites are indicated. The probe used for RNA gel blot analysis of alternatively polyadenylated transcripts is indicated in red [33]. (C) RNA gel blot analysis of IBM1 alternatively polyadenylated transcripts. β-TUBULIN (β-TUB.) was used as an internal control. (D) Reads mapping to At1g56500. Promoter proximal ‘P’ and distal ‘D’ alternative poly(A) sites are indicated. The probe used for RNA gel blot analysis of alternatively polyadenylated transcripts is indicated by a red line. (E) The predicted protein domain organisation encoded by At1g56500 mRNAs cleaved and polyadenylated at promoter proximal and distal poly(A) sites. At1g56500 encodes a putative hydrolase, but RNA cleavage at the proximal site eliminates a sequence encoding the putative TlpA-like TRX domain and three NHL (NCL-1, HT2A and LIN-41) repeats, while the sequence encoding the hydrolase domain (HAD-like) is retained. (F) RNA gel blot analysis of At1g56500 alternatively polyadenylated transcripts. β-TUBULIN (β-TUB.) was used as an internal control. Quantification of the bands revealed that At1g56500 polyadenylation at the distal and proximal sites is 18% and 82%, respectively, in fpa-7; 15% and 85% in fpa-7 flc-3; and 30% and 70% in wild-type (WT). (G) Reads mapping to At5g35170. Promoter proximal ‘P’ and distal ‘D’ alternative poly(A) sites are indicated. The probe used for RNA gel blot analysis of alternatively polyadenylated transcripts is indicated by a red line. (H) The predicted protein domain organisation encoded by At5g35170 mRNAs cleaved and polyadenylated at promoter proximal and distal poly(A) sites. At5g35170 encodes a putative adenylate kinase, but RNA cleavage at the proximal site eliminates a domain of unkn [file pgen.1003867.s004.pdf]

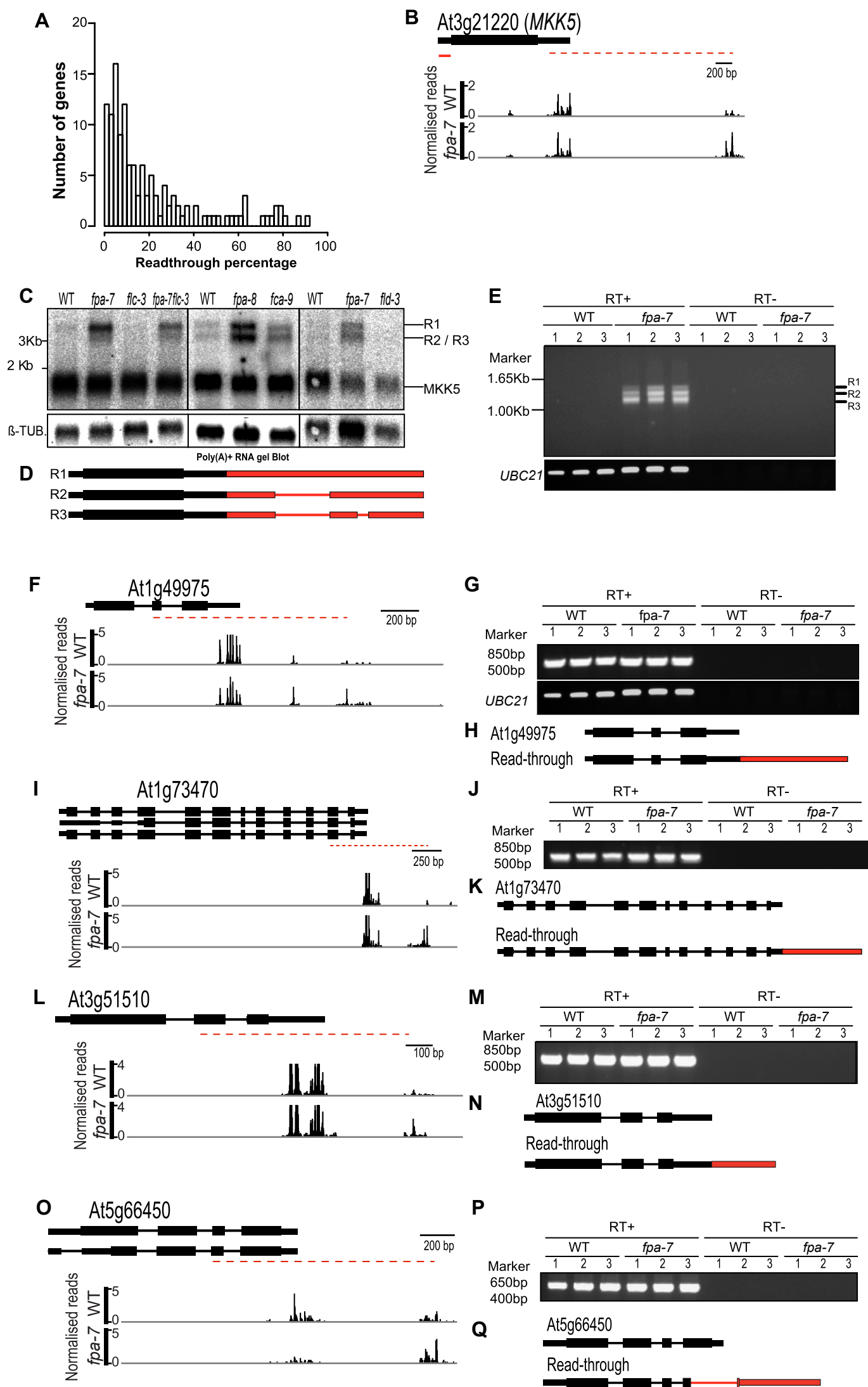

Figure S5. Characterization of intergenic read-through RNAs in *fpa* mutants.

Supplement: Figure S5 — FPA affects intergenic read-through. (A) Histogram of the read-through percentage in fpa-7 for protein-coding genes with differentially expressed downstream intergenic regions. (B-E) Characterisation of MKK5 intergenic read-through RNAs. (B) Normalised reads mapping to the MKK5 locus. The red dashed line indicates the region amplified by RT-PCR. (C) RNA gel blot analysis of MKK5 read-through transcripts. The probe used is indicated by a red solid line in (B). (D) Transcripts are either cleaved and polyadenylated in the annotated 3′UTR or at the intergenic sites, as determined by sequencing the cloned RT-PCR products. Red rectangles represent the 3′UTR specific to the read-through transcript and red lines represent 3′UTR introns. (E) Identification of contiguous RNA from the MKK5 gene upstream to the intergenic cleavage sites. RT-PCR products were separated on agarose gels and stained with ethidium bromide. Amplification controls for genomic DNA contamination (RT-) are included. Three biological replicates (1, 2 and 3) were used for each genotype: wild-type (WT) and fpa-7. (F–Q) Selection of tested read-through RNAs. (F, I, L, O) Normalised reads mapping to the analysed loci. Red dashed lines indicate the region amplified by RT-PCR. Images of normalised read alignments were made using the Integrated Genome Browser [55] and correspond to combined reads from the three sequenced biological replicates for each genotype. (G, J, M, P) Identification of contiguous RNAs from the upstream gene to the intergenic poly(A) sites. RT-PCR products were separated on agarose gels and stained with ethidium bromide. Amplification controls for genomic DNA contamination (RT-) are included. Three biological replicates (1, 2 and 3) were used for each genotype: WT and fpa-7. (H, K, N, Q) Transcripts are either cleaved and polyadenylated in the annotated 3′UTR or at the intergenic sites, as determined by sequencing the cloned RT-PCR products. Narrower red rectangles represent 3′UTRs specific [file pgen.1003867.s005.pdf]

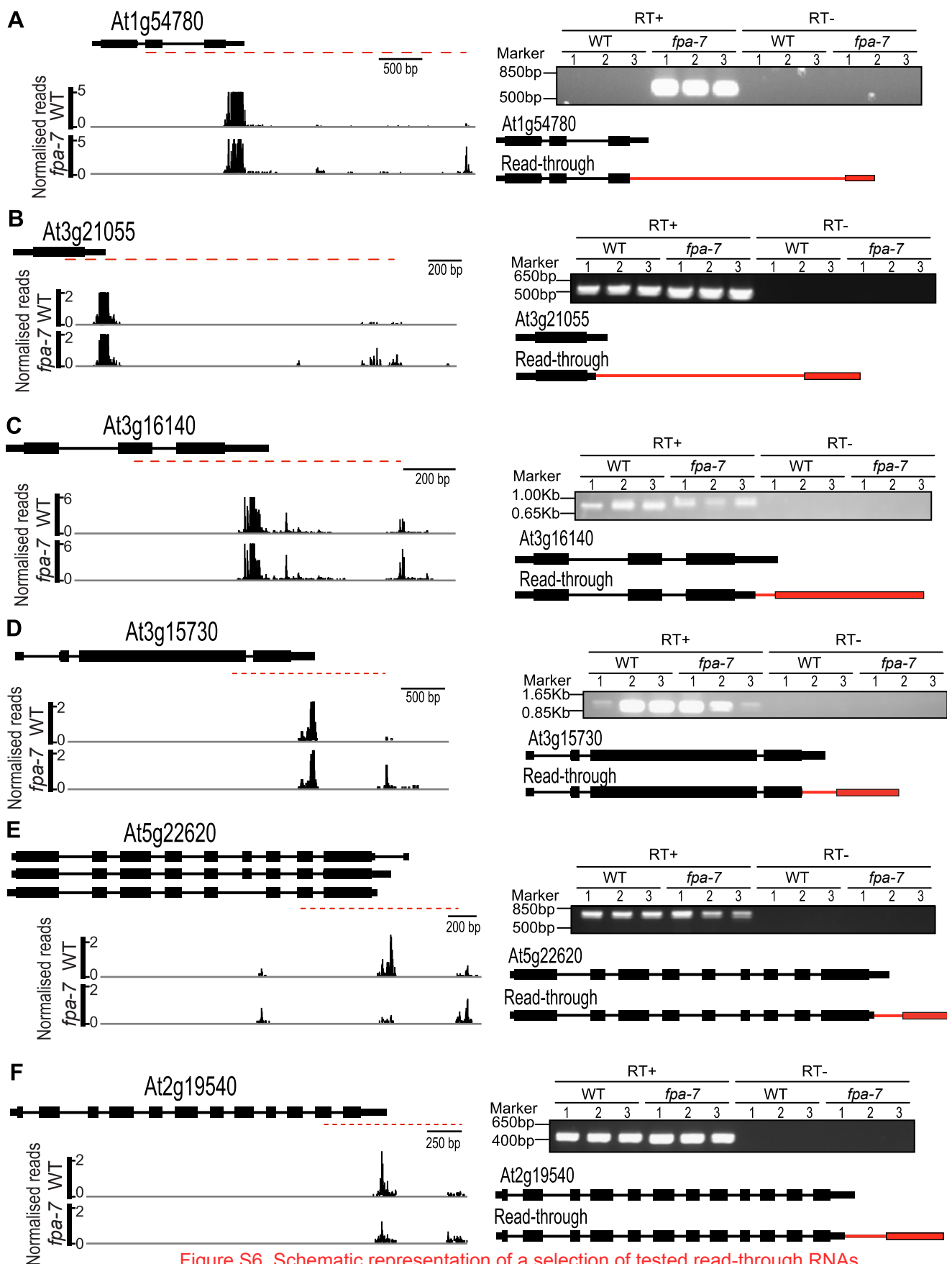

Figure S6. Schematic representation of a selection of tested read-through RNAs.

Supplement: Figure S6 — Schematic representation of a selection of tested read-through RNAs. (A–F) Left panels represent normalised reads mapping to the analysed loci. Red dashed lines indicate the regions amplified by RT-PCR. Images of normalised read alignments were made using the Integrated Genome Browser [55] and correspond to combined reads from the three sequenced biological replicates for each genotype. Top right panels show the identification of contiguous RNA from the upstream gene to the intergenic cleavage sites. RT-PCR products were separated on agarose gels and stained with ethidium bromide. Amplification controls for genomic DNA contamination (RT-) are included. Three biological replicates (1, 2 and 3) were used for each genotype: wild-type (WT) and fpa-7. Bottom right panels schematically show transcripts either cleaved and polyadenylated in the annotated 3′UTR or at the intergenic sites, as determined by sequencing the cloned RT-PCR products. Narrower red rectangles represent the 3′UTR section specific to the read-through transcript and red lines indicate the 3′UTR introns. (PDF) [file pgen.1003867.s006.pdf]

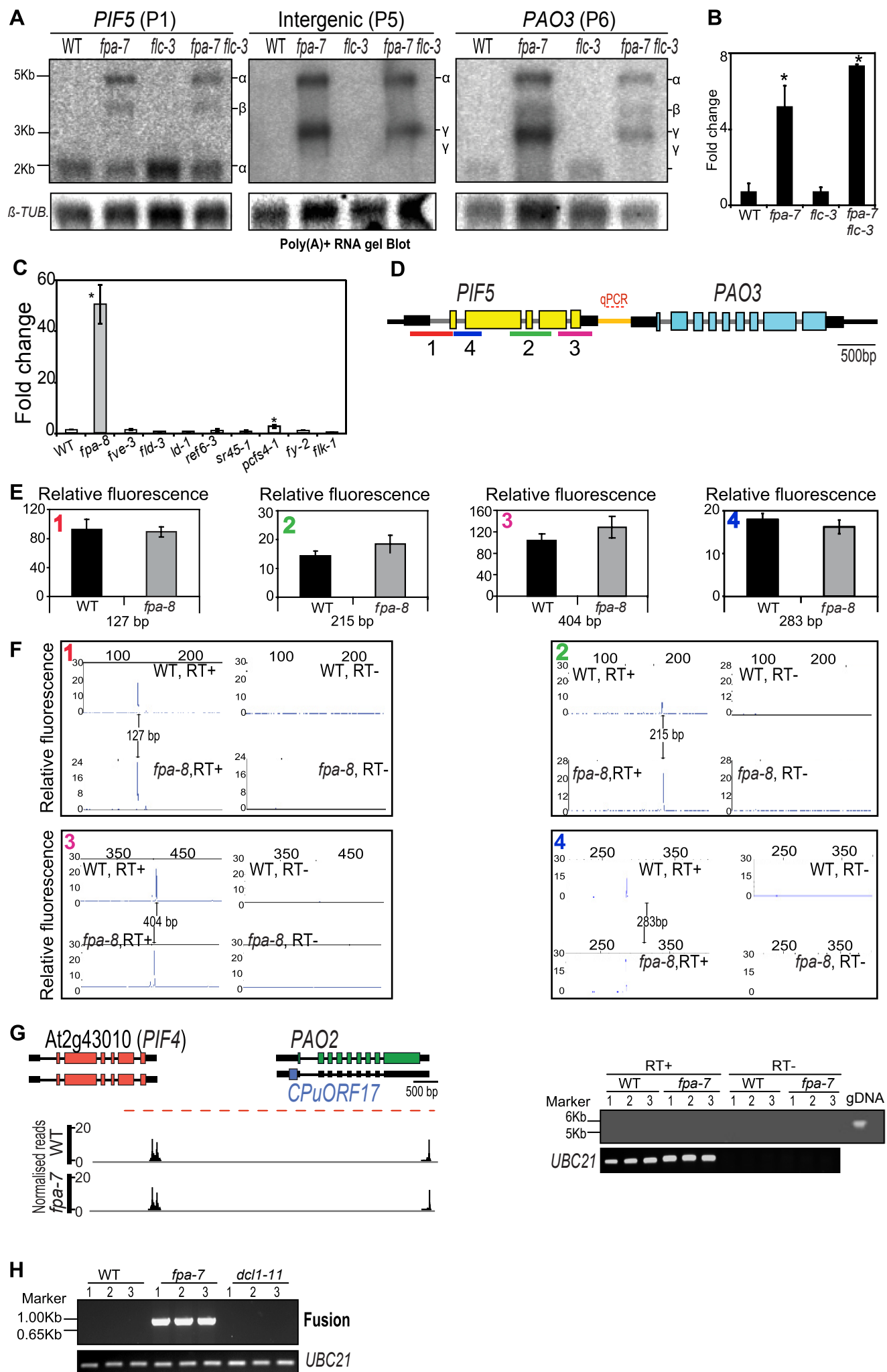

Figure S7. Analysis of potential causes of PIF5-PAO3 chimeric RNA formation.

Supplement: Figure S7 — Analysis of the potential causes of PIF5–PA03 chimeric RNA formation. (A) RNA gel blot analysis of PIF5–PA03 chimeric RNAs in fpa and flc mutants. P, PIF5; C, PA03 transcripts. β-TUBULIN (β-TUB.) was used as an internal control. Probes used are shown in Figure 5B . (B,C) RT-qPCR analysis of PIF5–PA03 chimeric RNAs in fpa and flc (B) and late-flowering (C) mutants. Location of the RT-qPCR amplicon is displayed in (D) by a red dotted line and labelled qPCR. PIF5–PA03 chimeric RNAs were quantified by RT-qPCR analysis of the expression of the intergenic fragment between PIF5 and PA03. Data are the means ± SEM obtained for three independent PCR amplifications of three biological replicates. The y-axis shows the fold change relative to wild-type (WT; set to 1) after normalisation to UBC21 gene expression. *, P<0.05; Student's t-test. (D) Structure of the PIF5–PA03 locus: exons are denoted by yellow rectangles, UTRs by narrower black rectangles and introns by grey lines; regions analysed for splicing efficiency and accuracy are indicated (1, red; 2, green; 3, pink; and 4, blue). The red dashed line indicates the location of the RT-qPCR amplicon. (E) Expression levels of PIF5 spliced RNA for primer sets 1, 2, 3 and 4; their location is displayed in (D). Histograms show means ± SEM obtained from three PCR reactions for each of three biological replicates. (F) Electropherograms for primer sets 1, 2, 3 and 4. The forward primer in each set was 6-FAM labelled. Numbers on the x-axis represent size markers in nucleotides; numbers on the y-axis represent relative fluorescence ×103, reflecting transcript abundance. Electropherograms are representative of three biological and three PCR replicates. The main splicing product is identified by its size. No significant changes in the ratios of alternatively spliced transcripts were observed between WT and fpa-8. (G) Normalised reads mapping to loci encoding PIF4 and PA02. Images of normalised read alignments on the left panel were made u [file pgen.1003867.s007.pdf]

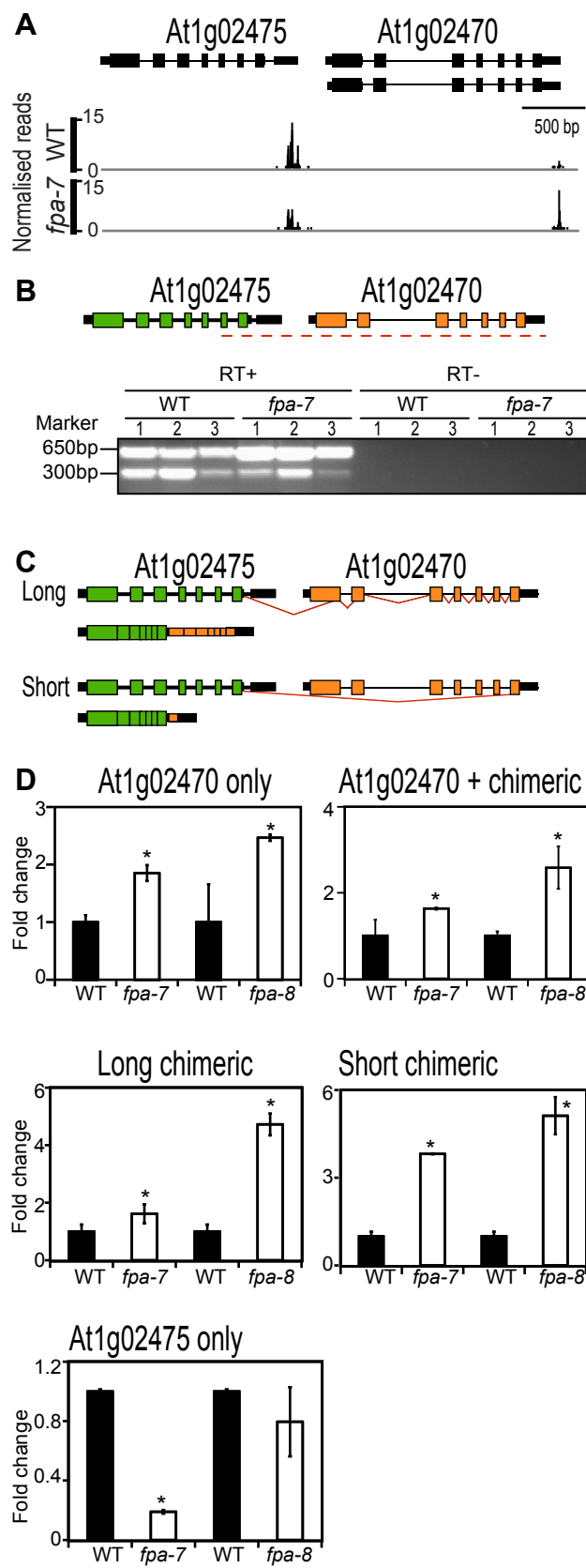

Figure S8. Analysis of chimeric RNAs formed between At1g02475 and At1g02470.

Supplement: Figure S8 — Analysis of chimeric RNAs formed between At1g02475 and At1g02470. (A) Normalised reads mapping to the At1g02475–At1g02470 loci. Exons are denoted by rectangles, UTRs by adjoining narrower rectangles and introns by lines. Images of normalised read alignments were made using the Integrated Genome Browser [55] and correspond to combined reads from the three sequenced biological replicates for each genotype. (B) Chimeric RNAs identified by RT-PCR. The region amplified by RT-PCR is indicated by a red dashed line. RT-PCR products were separated on agarose gels and stained with ethidium bromide. Amplification controls for genomic DNA contamination (RT-) are included. Three biological replicates (1, 2 and 3) were used for each genotype: wild-type (WT) and fpa-7. (C) Schematic representation of the chimeric RNAs, as determined by sequencing the cloned RT-PCR products. A red line depicts splicing events. (D) RT-qPCR analysis of the different RNAs expressed at the At1g02475–At1g02470 loci in fpa mutants. Data are the means ± SEM obtained for three independent PCR amplifications on three biological replicates. The y-axis shows the fold change relative to wild-type (set to 1) after normalisation to UBC21 gene expression. (PDF) [file pgen.1003867.s008.pdf]

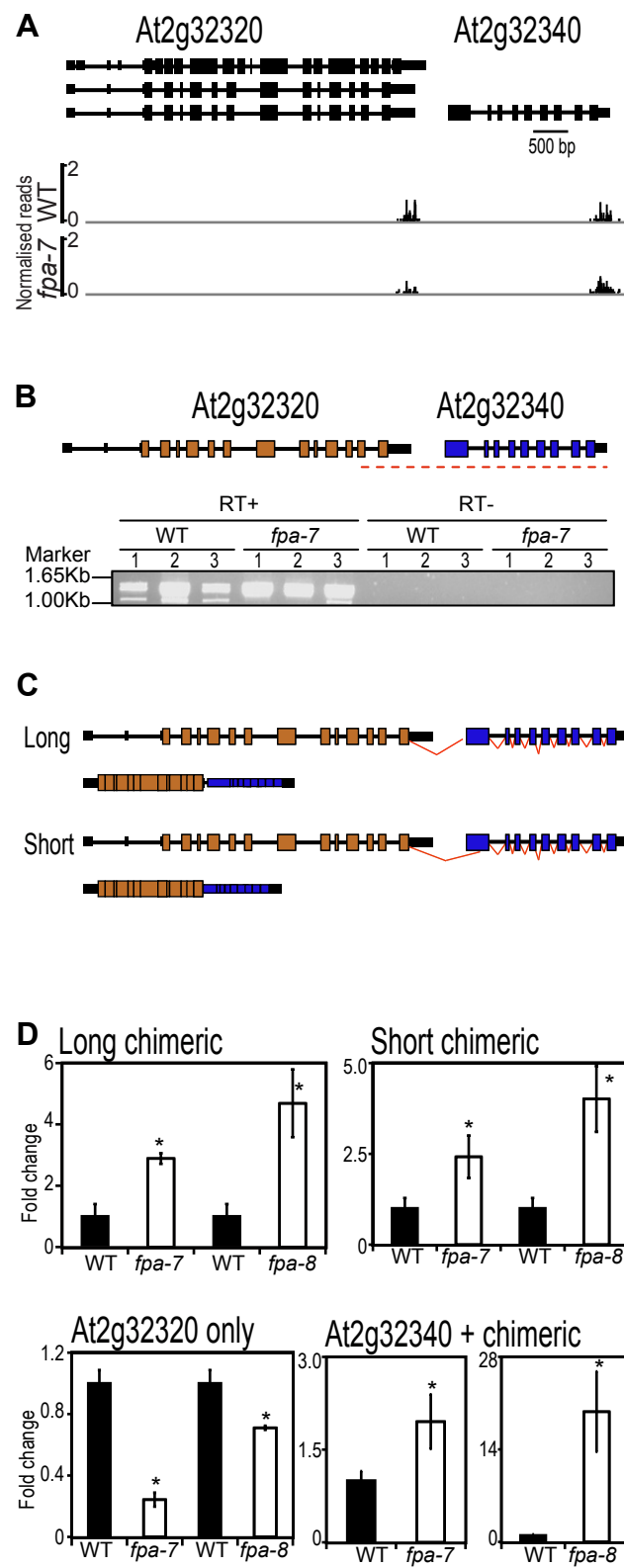

Figure S9. Analysis of chimeric RNAs formed between At2g32320 and At2g32340.

Supplement: Figure S9 — Analysis of chimeric RNAs formed between At2g32320 and At2g32340. (A) Normalised reads mapping to the At2g32320–At2g32340 loci. Exons are denoted by rectangles, UTRs by adjoining narrower rectangles and introns by lines. Images of normalised read alignments were made using the Integrated Genome Browser [55] and correspond to combined reads from the three sequenced biological replicates for each genotype. (B) Chimeric RNAs identified by RT-PCR. The region amplified by RT-PCR is indicated by a red dashed line. RT-PCR products were separated on agarose gels and stained with ethidium bromide. Amplification controls for genomic DNA contamination (RT-) are included. Three biological replicates (1, 2 and 3) were used for each genotype: wild-type (WT) and fpa-7. (C) Schematic representation of the chimeric RNAs, as determined by sequencing the cloned RT-PCR products. A red line depicts splicing events. (D) RT-qPCR analysis of the different RNAs expressed at the At2g32320–At2g32340 locus in fpa mutants. Data are the means ± SEM obtained for three independent PCR amplifications of three biological replicates. The y-axis shows the fold change relative to WT (set to 1) after normalisation to UBC21 gene expression. Chimeric RNAs formed between At2g32320 and At2g32340 did not alter the coding potential of the upstream gene but changed the effective 3′UTR. (PDF) [file pgen.1003867.s009.pdf]

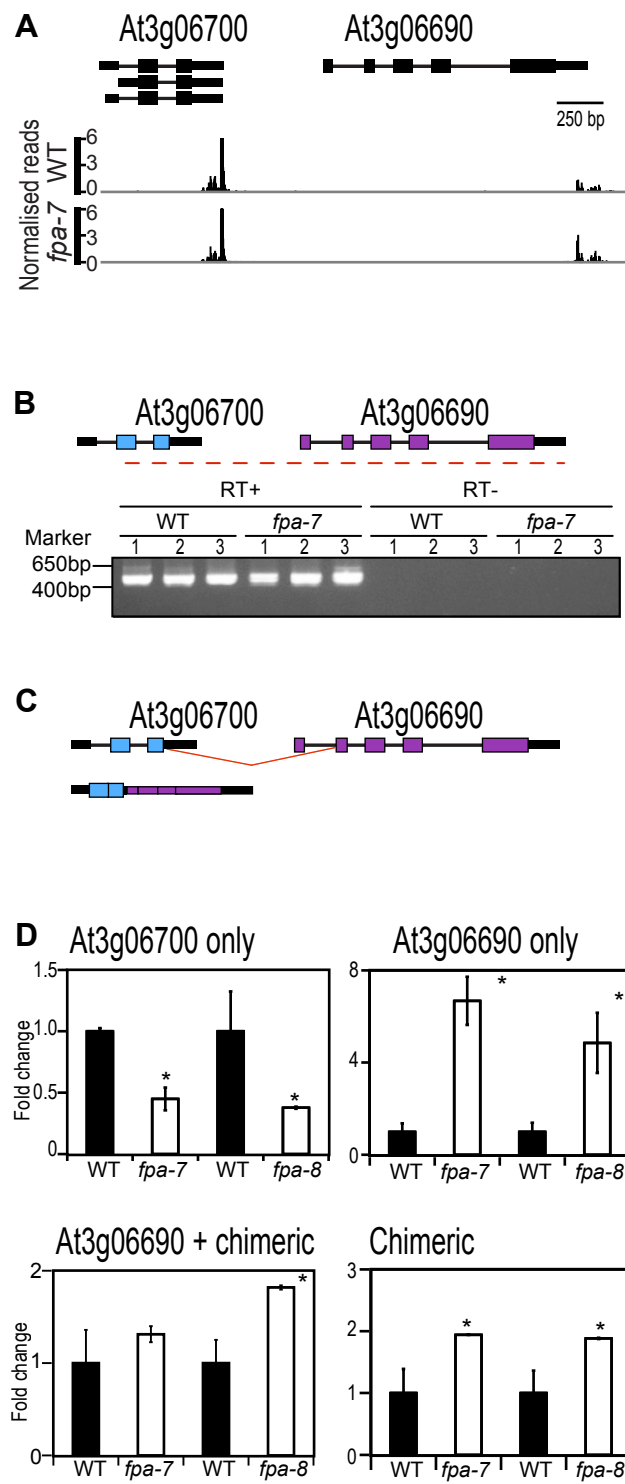

Figure S10. Analysis of chimeric RNAs formed between At3g06690 and At3g06700.

Supplement: Figure S10 — Analysis of chimeric RNAs formed between At3g06700 and At3g06690. (A) Normalised reads mapping to the At3g06700–At3g06690 loci. Exons are denoted by rectangles, UTRs by adjoining narrower rectangles and introns by lines. Images of normalised read alignments were made using the Integrated Genome Browser [55] and correspond to combined reads from the three sequenced biological replicates for each genotype. (B) Chimeric RNAs identified by RT-PCR. The region amplified by RT-PCR is indicated by a red dashed line. RT-PCR products were separated on agarose gels and stained with ethidium bromide. Amplification controls for genomic DNA contamination (RT-) are included. Three biological replicates (1, 2 and 3) were used for each genotype: wild-type (WT) and fpa-7. (C) Schematic representation of the chimeric RNAs, as determined by sequencing the cloned RT-PCR products. A red line depicts splicing events. (D) RT-qPCR analysis of the different RNAs expressed at the At3g06700–At3g06690 loci in fpa mutants. Data are the means ± SEM obtained for three independent PCR amplifications of three biological replicates. The y-axis shows the fold change relative to WT (set to 1) after normalisation to UBC21 gene expression. Chimeric RNAs formed between At3g06700 and At3g06690 did not alter the coding potential of the upstream gene but changed the effective 3′UTR. (PDF) [file pgen.1003867.s010.pdf]

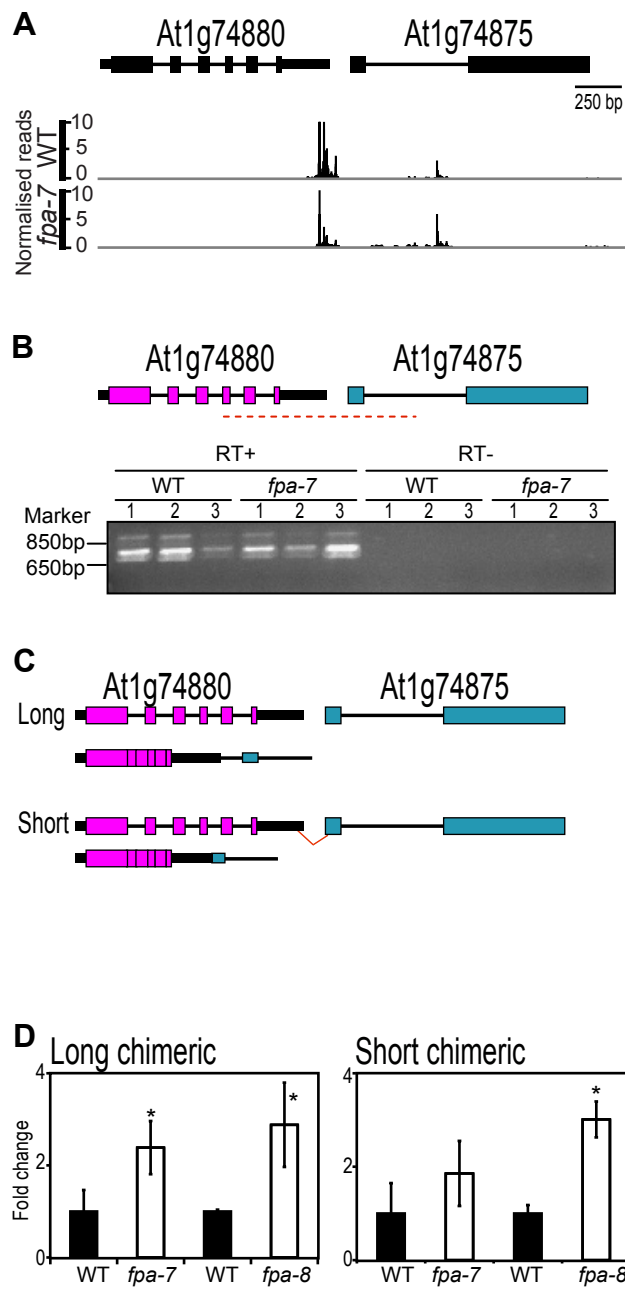

Figure S11. Analysis of chimeric RNAs formed between At1g74880 and At1g74875.

Supplement: Figure S11 — Analysis of putative chimeric RNAs formed between At1g74880 and At1g74875. (A) Normalised reads mapping to the At1g74880–At1g74875 locus. Exons are denoted by rectangles, UTRs by adjoining narrower rectangles and introns by lines. Images of normalised read alignments were made using the Integrated Genome Browser [55] and correspond to combined reads from the three sequenced biological replicates for each genotype. (B) Chimeric RNAs were identified by RT-PCR. The region amplified by RT-PCR is indicated by a red dashed line. RT-PCR products were separated on agarose gels and stained with ethidium bromide. Amplification controls for genomic DNA contamination (RT-) are included. (C) Schematic representation of the chimeric RNAs, as determined by sequencing of cloned RT-PCR products. A red line depicts splicing events. (D) RT-qPCR analysis of the different RNAs expressed at the At1g74880–At1g74875 locus in fpa mutants. Data are the means ± SEM obtained for three independent PCR amplifications on three biological replicates. The y-axis shows the fold change relative to wild-type (WT; set to 1) after normalisation to UBC21 gene expression. The significance of this validated example of chimeric RNA formation (At1g74875 and At1g74880) is less clear because the downstream gene is relatively poorly characterised; it may reflect annotation errors rather than chimeric RNA formation between two well-characterised genes. (PDF) [file pgen.1003867.s011.pdf]

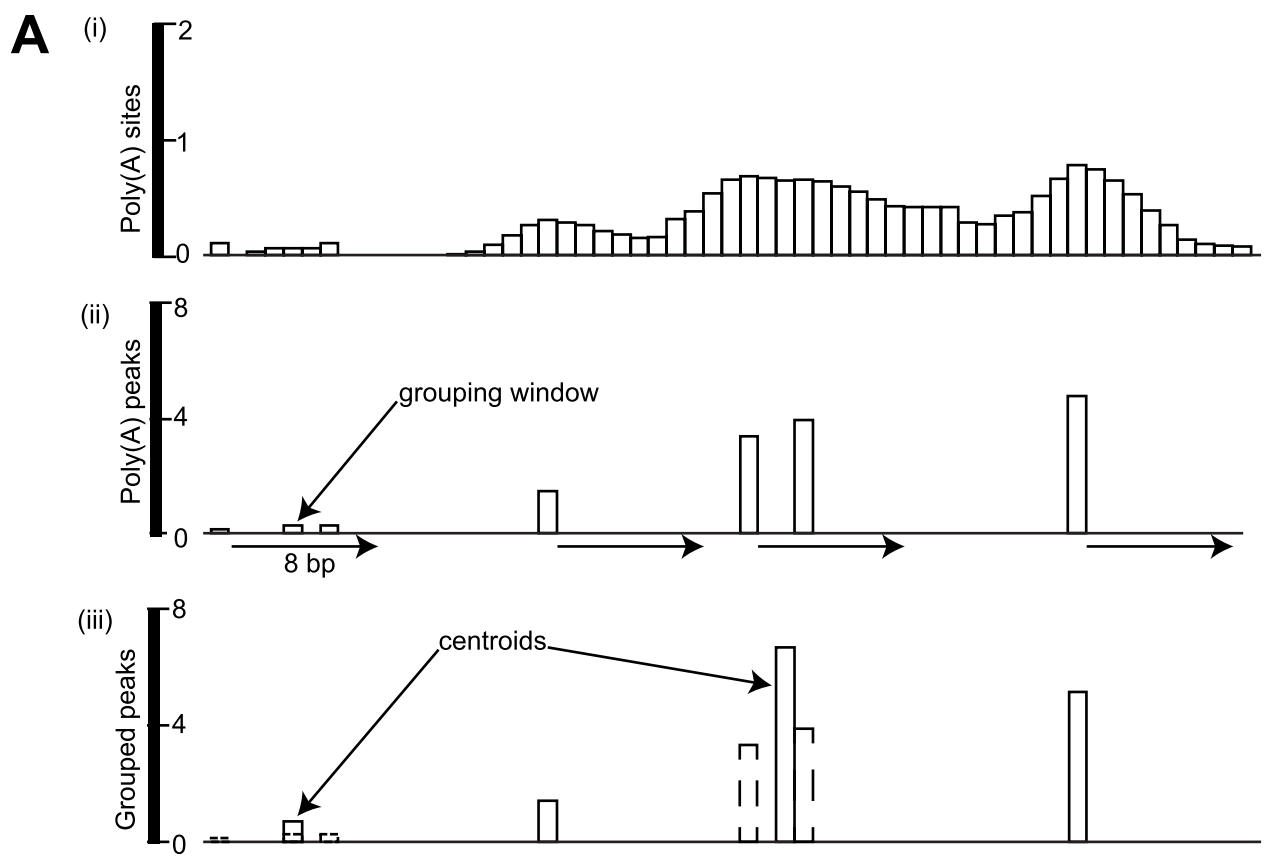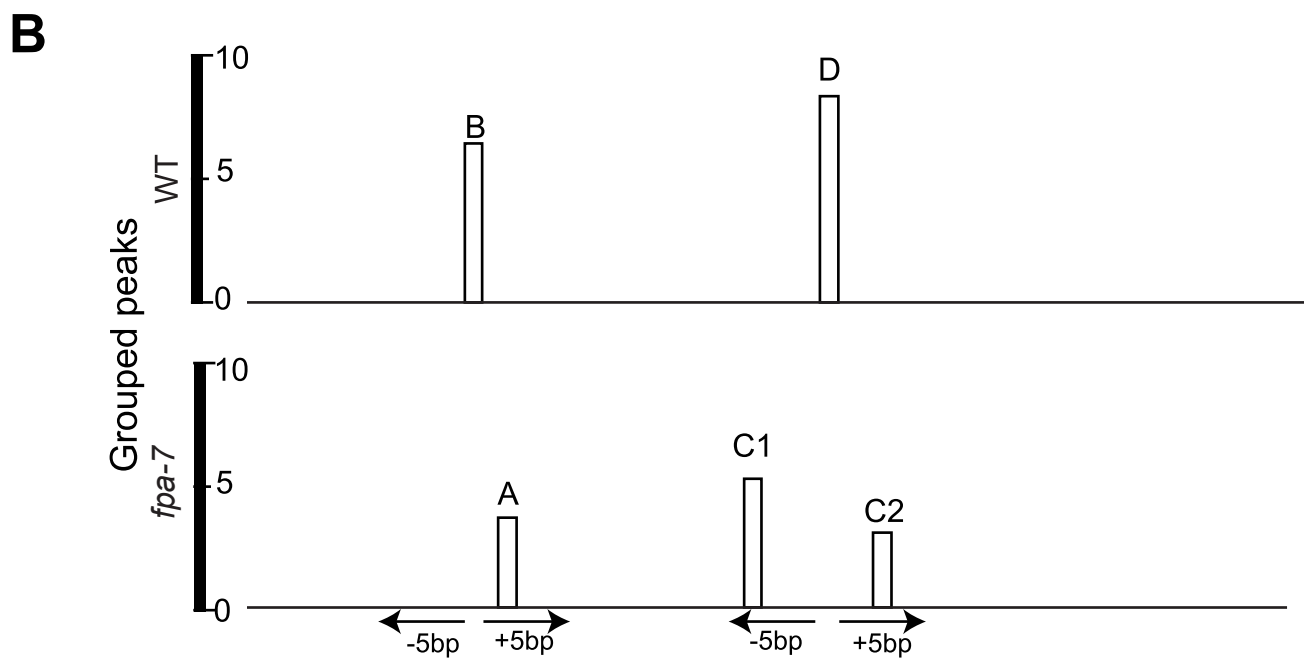

Figure S12. Illustration of peak grouping and peak matching algorithms.

Supplement: Figure S12 — Illustration of the peak combination and peak-matching algorithms. (A) Grouping algorithm: (i) stylised smoothed read profile; (ii) peaks called by our algorithm as previously described [1]–[3], [22] (ii) peaks that are close together are often due to errors in peak calling and so are combined as follows. For each peak, a search is made in the 8-bp sequence 3′ of the peak. If a further peak is found, then the two peaks are combined, with a new peak being established at the centroid of the two positions. The value of 8 bp was chosen by experimentation. (B) Peak-matching algorithm: for each peak in wild-type (WT), an equivalent peak in fpa-7 is identified if it is within +/−5 bp. Peaks A and B were matched. If two peaks are found, then the first is taken as the equivalent peak and the second is assigned to zero. Peaks C1 and D were matched; peak C2 is matched with 0 in WT. Images of normalised read alignments were made using the Integrated Genome Browser [55] and correspond to combined reads from the three sequenced biological replicates for each genotype. (PDF) [file pgen.1003867.s012.pdf]

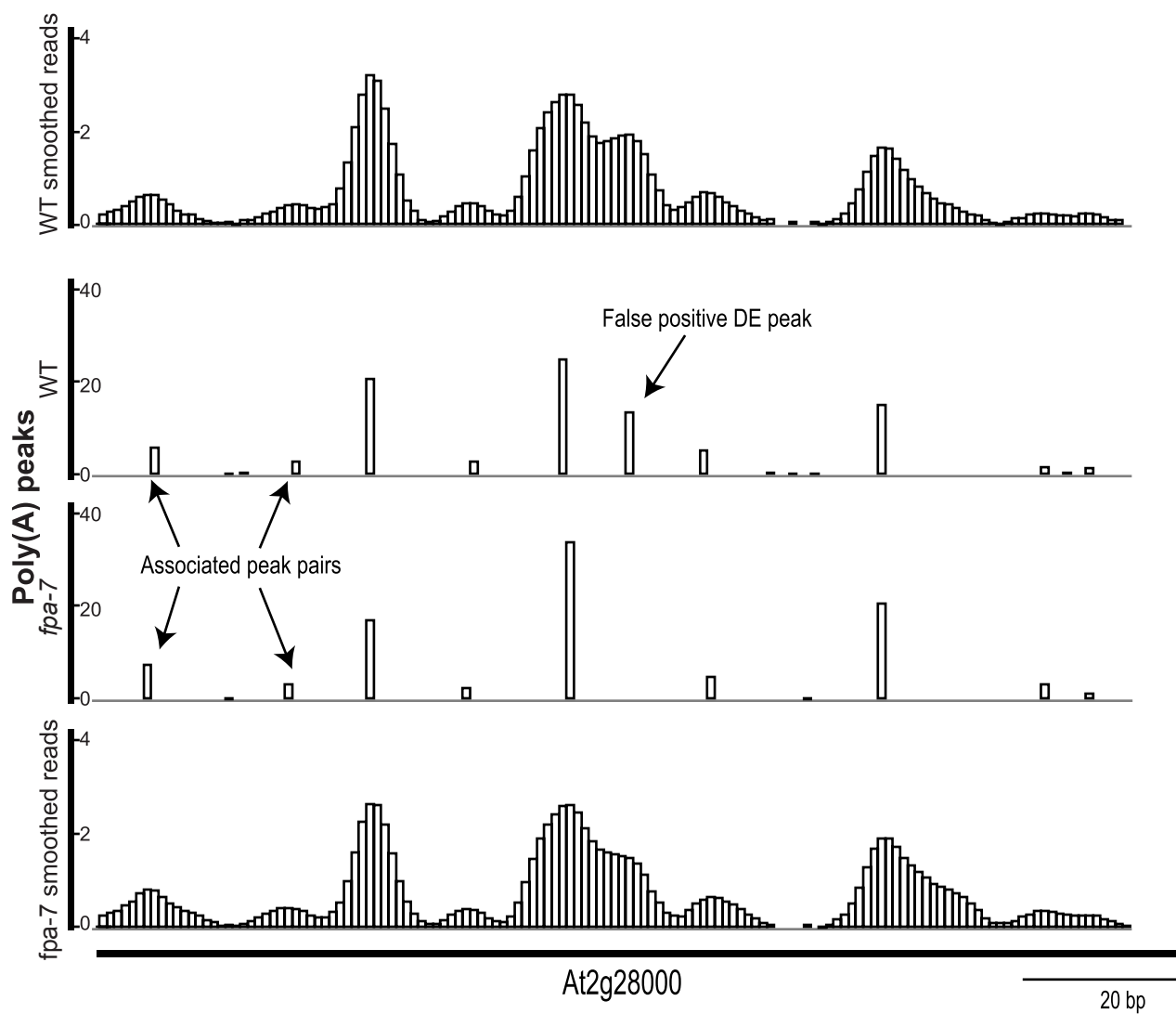

Figure S13. Example of “false positives” and associated poly(A) peaks at *At2g28000*.

Supplement: Figure S13 — Example of a ‘false positive’ differentially expressed poly(A) peak and matched poly(A) peaks at At2g28000. Top and bottom panels, smoothed raw poly(A) sites mapping to At2g28000; middle panels, poly(A) peaks called from these smoothed poly(A) profiles. All data were subject to read-per-million (RPM) normalisation. Images of normalised read alignments were made using the Integrated Genome Browser [55] and correspond to combined reads from the three sequenced biological replicates for each genotype. (PDF) [file pgen.1003867.s013.pdf]
