# Supplementary material for: Transcription Termination and Chimeric RNA Formation Controlled by Arabidopsis thaliana FPA
Source: PLoS Genet. 2013 Oct 31;9(10):e1003867. doi: 10.1371/journal.pgen.1003867 (PMC3814327; doi:10.1371/journal.pgen.1003867)
Supplement: Figure S3 — FPA-dependent alternative polyadenylation events. (A) Location of the poly(A) sites ‘T’ due to the insertion in fpa-7. (B) Additional cleavage sites found within intron 4 upstream of the fpa-7 T-DNA insertion. DRS reads that map to fpa-7 T1 sites are displayed. The positions of 3′RACE products are indicated above the DNA sequence by red triangles (numbers indicate how many 3′RACE clones were obtained). Images of normalised read alignments were made using the Integrated Genome Browser [55] and correspond to combined reads from the three sequenced biological replicates for each genotype. (C) Additional cleavage sites found within intron 5 upstream of the fpa-7 T-DNA insertion. DRS reads that map to fpa-7 T2 sites are displayed. Positions of 3′RACE products are indicated on the DNA sequence by red triangles (the numbers indicate how many 3′RACE clones were obtained). (D) Additional cleavage sites found within intron 5 upstream of the fpa-7 T-DNA insertion. Positions of 3′RACE products are marked above the DNA sequence by red triangles (the numbers indicate how many 3′RACE clones were obtained), while DRS reads that aligned to the FPA T-DNA junction are marked by blue triangles (with the number of raw reads shown above). A black line indicates the T-DNA sequence. (E) 3′RACE analysis of the mom1–3 mutant. Positions of 3′RACE products are marked on the DNA sequence by red triangles (numbers indicate how many 3′RACE clones were obtained). As the T-DNA mutant mom1–3 also displayed cryptic cleavage sites upstream of its T-DNA insertion, it is possible that premature cleavage and polyadenylation may form part of the mechanism by which T-DNAs disrupt gene function; this raises the general question of how cryptic sites are triggered. (F) Structure of the T-DNA. The left and right borders (‘LB’ and ‘RB’, respectively) are indicated by red boxes. NPTII is the kanamycin resistance gene used for selection. (PDF) [file pgen.1003867.s003.pdf]

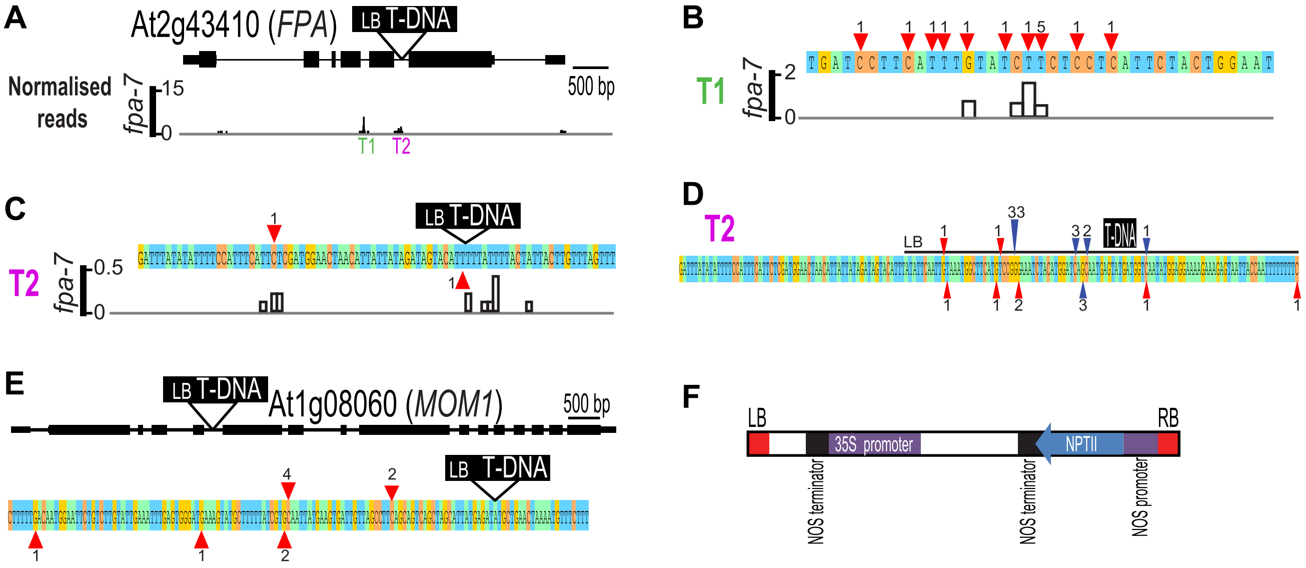

Figure S3. Analysis of alternatively polyadenylated transcripts.
